# Time to appropriate treatment in patients with multidrug-resistant tuberculosis in South Korea: Are we still in 2010?

**DOI:** 10.1371/journal.pone.0216084

**Published:** 2019-04-25

**Authors:** Eun-Jung Jo, Seyeon Park, Kyu Min Lee, Insu Kim, Jung Seop Eom, Mi-Hyun Kim, Kwangha Lee, Ki Uk Kim, Hye-Kyung Park, Min Ki Lee, Jeongha Mok

**Affiliations:** 1 Department of Internal Medicine, Pusan National University Hospital, Busan, Korea; 2 College of Medicine, Pusan National University, Yangsan, Korea; 3 Biomedical Research Institute, Pusan National University Hospital, Busan, Korea; The University of Georgia, UNITED STATES

## Abstract

**Background:**

This study investigated the time to appropriate treatment and factors affecting late treatment initiation in patients with multidrug-resistant tuberculosis (MDR-TB) in South Korea.

**Methods:**

Data from patients with culture-confirmed pulmonary MDR-TB who received treatment at Pusan National University Hospital (PNUH) between January 2010 and July 2018 were reviewed retrospectively. Patients were divided into two groups according to the first institution they visited [patients who were transferred to PNUH after diagnosis of MDR-TB (Group A) and patients who were initially diagnosed with TB at PNUH (Group B)].

**Results:**

A total of 100 patients were included (53 in Group A and 47 in Group B). The percentage of patients in whom line probe assays (LPAs) for isoniazid and rifampin or Xpert MTB/RIF assays were performed was higher in Group B than in Group A [20.8 *vs*. 57.4% (*P* < 0.001) and 17.0 *vs*. 46.8% (*P* = 0.001), respectively]. The median time from the first visit to appropriate treatment initiation was longer in Group A (102.0 *vs*. 77.0 days, *P* = 0.002). However, a subgroup analysis of patients with pre-extensively or extensively drug-resistant TB (pre-XDR- or XDR-TB) revealed that the time to appropriate treatment did not differ between Groups A and B. Although the time to appropriate treatment decreased during the study period in both Groups A and B, this trend was not evident in patients with pre-XDR- or XDR-TB in Group B. Based on multivariate analyses, performance of LPAs for isoniazid and rifampin, performance of Xpert MTB/RIF assays, and the presence of uncomplicated MDR-TB were protective against delays in appropriate treatment initiation.

**Conclusions:**

The time to appropriate treatment in patients with MDR-TB in South Korea was not acceptable, particularly for patients diagnosed outside of PNUH and for patients with pre-XDR- or XDR-TB. The use of rapid molecular drug susceptibility tests in various healthcare settings and introduction of second-line LPAs are required.

## Introduction

Multidrug-resistant tuberculosis (MDR-TB) is a major public health problem worldwide that complicates control and elimination of TB [1,2]. Treatment for MDR-TB is prolonged, as second-line drugs are less effective and more toxic than first-line drugs [3]. Thus, treatment outcomes are often unsatisfactory. In a 2015 global MDR-TB cohort, only 55% of patients successfully completed treatment [2]. The treatment outcomes of patients with extensively drug-resistant TB (XDR-TB) were poor because only 34% of patients completed treatment successfully [2].

Early diagnosis and initiation of effective therapy are essential for positive treatment outcomes in patients with MDR-TB [4–6]. Delayed diagnosis and treatment may result in an increased bacillary load and drug resistance, extensive lung damage, and treatment failure or death [6–11]. In addition, inappropriate treatment before the diagnosis of MDR-TB may increase the risk of transmission of drug-resistant strains and allow new MDR-TB patients to emerge in the community [12–15]. However, a systematic review reported delays in the diagnosis and treatment of patients with MDR-TB [9].

In South Korea, diagnosis and treatment of MDR-TB are often delayed. A Korean study conducted in the early 2000s revealed that the interval from sputum collection to a confirmed diagnosis of MDR-TB by culture-based phenotypic drug susceptibility testing (DST) was about 3 months [16]. Since the early 2010s, there have been many changes regarding diagnosis of MDR-TB in South Korea. New rapid molecular DSTs, such as line probe assays (LPAs) for isoniazid (INH) and rifampin (RIF) and Xpert MTB/RIF assays, have been introduced into clinical practice. The use of these assays has increased, especially in tertiary referral hospitals and designated TB care centers. However, no study has explored how these changes have affected time to treatment in patients with MDR-TB in South Korea.

In this study, time to appropriate treatment in patients with MDR-TB in South Korea was evaluated according to the first institution visited. Factors delaying treatment initiation and treatment outcomes in patients with MDR-TB were also evaluated.

## Methods

### Study design and subjects

This study was reviewed and approved by the Institutional Review Board of Pusan National University Hospital (PNUH; approval number: H-1812-018-074). The need for informed consent from patients was waived due to the observational and retrospective nature of the study. The study had no impact on diagnosis or treatment of patients. Data were de-identified prior to analysis.

This retrospective cohort study was conducted at PNUH, a university-affiliated tertiary care hospital in Busan, South Korea, with 1400 beds. The incidence rates of total and new TB cases in South Korea were 96.4 and 72.8 per 100,000 population, respectively, in 2010 and 70.4 and 55.0 per 100,000 population in 2017 [17]. In 2017, 3.2% of newly diagnosed and 10.0% of recurring cases were MDR-TB [2]. PNUH is well-equipped for diagnosis and treatment of TB (e.g., pulmonology/TB specialists, advanced laboratory facilities, infrastructures such as negative pressure rooms, and participation in Public-Private Mix collaborations).

Culture-confirmed pulmonary MDR-TB patients who initiated treatment at PNUH between January 2010 and July 2018 were enrolled. Patients with extrapulmonary TB only or patients whose medical records did not indicate the first institution visited before transfer to PNUH were excluded from the analysis. Patients were divided into two groups according to the first institution they visited for TB diagnosis. Group A included patients who were transferred to PNUH after MDR-TB diagnosis [including patients with rifampin-resistant (RR)-TB on Xpert MTB/RIF assay], and Group B included patients who were initially diagnosed with TB at PNUH.

### Data collection

The following data were collected from the medical records: age, sex, height, body weight, comorbidities, smoking status, previous treatment history for TB, initial sputum acid fast bacilli (AFB) smear and phenotypic DST results, and chest computed tomography findings. The diagnostic modalities performed for each patient and their results were also collected (i.e., bronchoscopy, LPA for INH and RIF, and/or Xpert MTB/RIF assay). The first institution visited, the date of the first visit, the date of and reason for transfer to PNUH (for Group A), and appropriate treatment dates were also evaluated. The use of and duration of treatment with first-line anti-TB drugs, the date of sputum AFB smear and *Mycobacterium tuberculosis* culture conversion, and final treatment outcomes were evaluated.

### Definitions

MDR-TB was defined as TB that was resistant to both INH and RIF. XDR-TB was defined as MDR-TB that was further resistant to any fluoroquinolone (FQ) and at least one of the three second-line injectable drugs (SLIDs; kanamycin, amikacin, and capreomycin). Pre-XDR-TB was defined as MDR-TB that was further resistant to either an FQ or any SLID but not both. Uncomplicated MDR-TB was defined as MDR-TB without additional resistance to FQ or any SLID. Patients were classified into two groups according to their TB treatment history: new patients who had never been treated for TB or who had taken anti-TB drugs for < 1 month, and previously treated patients who had received anti-TB drugs for ≥ 1 month [18].

Time to appropriate treatment was defined as the time from the first visit for TB to the initiation of appropriate MDR-TB treatment. Sputum smear conversion and culture conversion were defined as two consecutive negative results collected at least 30 days apart from a patient with a positive specimen at baseline. The time of sputum smear or culture conversion was defined as the day of sample collection for the first of the two consecutive negative results. Patients were categorized on the basis of treatment outcome in accordance with World Health Organization (WHO) definitions as follows: cured, treatment completed, treatment failed, died, lost to follow-up, and not evaluated [18]. The rate of treatment success included patients who were cured and those who completed treatment.

### Drug susceptibility tests and MDR-TB treatment

For phenotypic DSTs, all *M*. *tuberculosis* isolates were sent to the Korean Institute of Tuberculosis. The susceptibility of the *M*. *tuberculosis* isolates to the following drugs was determined using the absolute concentration method with Lowenstein-Jensen medium: INH, RIF, ethambutol, rifabutin, streptomycin, amikacin, kanamycin, capreomycin, ofloxacin, levofloxacin, moxifloxacin, prothionamide, cycloserine, and para-aminosalicylic acid. Pyrazinamide susceptibility was determined using a pyrazinamidase test. The overall workflow for the phenotypic DSTs did not differ between Group A and B.

LPAs for INH and RIF (GenoType MTBDR*plus*; Hain Lifescience GmbH, Nehren, Germany or AdvanSure MDR-TB GenoBlot assay; LG Life Sciences, Seoul, Korea) and the Xpert MTB/RIF assay (Cepheid, Sunnyvale, CA, USA) were introduced in South Korea in 2007 and 2012, respectively. All tests were performed according to the manufacturer`s instructions. Rapid molecular DSTs included both LPAs for INH and RIF and the Xpert MTB/RIF assay. MDR- or RR-TB were confirmed using either LPAs for INH and RIF or the Xpert MTB/RIF assay.

MDR-TB treatment regimens were individualized based on the phenotypic DST results and guided by WHO recommendations (at least four effective second-line anti-TB drugs with or without pyrazinamide for at least 18–20 months) [19–21]. All MDR-TB treatment regimens included an FQ and a SLID if there was no resistance or intolerance to these drugs. All patients started MDR-TB treatment in negative pressure rooms under forced hospitalization and were discharged from hospital only after the infectious period had passed. This has been a feature of the National TB Program since 2012. The anti-TB drugs were administered under direct observation during hospitalization and subsequently self-administered after discharge.

### Statistical analysis

Continuous variables are given as median with interquartile ranges, and categorical variables are given as numbers or percentages. Continuous variables were compared using independent t-tests and categorical variables were compared using Pearson’s chi-square test or Fisher’s exact test. Linear regression analysis was performed to assess annual trends in time to appropriate treatment, and chi-square tests were used to assess annual trends in the performance of LPAs for INH and RIF, or Xpert MTB/RIF assays. Univariate and multivariate logistic regression analyses were performed to evaluate factors affecting late initiation of appropriate treatment. A *P* value of < 0.05 was considered significant. Statistical analyses were performed using SPSS Statistics, version 22.0 (SPSS Inc., Chicago, IL, USA).

## Results

### Patient characteristics

A total of 111 patients were screened for inclusion during the study period. After applying the criteria outlined above, 100 patients were included in the analysis (three patients with extrapulmonary TB only and eight patients with medical records that did not include the first institution visited before transferring to PNUH were excluded; no difference was noted in the baseline characteristics of the included and excluded patients). Among the 100 patients, 53 (53.0%) were in Group A and 47 (47.0%) were in Group B. Table 1 lists the baseline characteristics of all patients. There were no differences between Group A and B in terms of clinical or demographic parameters. Semi-hospitals were the most common institution first visited by patients in Group A (n = 38, 71.7%), followed by general hospitals (n = 7, 13.2%), public health centers (n = 6, 11.3%), and clinics (n = 2, 3.8%). The reasons that Group A patients transferred to PNUH were as follows: diagnosis of MDR-TB by phenotypic DST (n = 40, 75.5%); diagnosis of MDR-TB by LPA for INH and RIF (n = 7, 13.2%); and diagnosis of RR-TB by the Xpert MTB/RIF assay (n = 6, 11.3%).

**Table 1:** Baseline patient characteristics.

	Total (n = 100)	Group A (n = 53)	Group B (n = 47)	*P* Value*
Age, years	45.0 [33.0–57.8]	45.0 [31.5–58.0]	47.0 [35.0–57.0]	0.555
Male sex	64 (64.0)	31 (58.5)	33 (70.2)	0.223
BMI, kg/m^2^	20.8 [18.8–23.4]	20.7 [18.6–23.6]	20.9 [19.4–23.4]	0.980
Comorbidity				
HIV infection	0 (0.0)	0 (0.0)	0 (0.0)	> 0.999
Diabetes mellitus	23 (23.0)	10 (18.9)	13 (27.7)	0.297
Hypertension	16 (16.0)	8 (15.1)	8 (17.0)	0.793
Malignancy	9 (9.0)	3 (5.7)	6 (12.8)	0.299
Chronic lung disease	5 (5.0)	2 (3.8)	3 (6.4)	0.664
Ever smoker	45 (45.0)	23 (43.4)	22 (46.8)	0.732
Previous treatment history for TB	39 (39.0)	20 (37.7)	19 (40.4)	0.783
Bilateral disease on chest CT	44 (44.0)	26 (49.1)	18 (38.3)	0.279
Cavity on chest CT	62 (62.0)	37 (69.8)	25 (53.2)	0.087
Sputum AFB smear (+)^†^	58 (58.0)	34 (64.2)	24 (51.1)	0.186
Resistance level				
Uncomplicated MDR^‡^	68 (68.0)	32 (60.4)	36 (76.6)	0.083
Pre-XDR with SLID resistance	10 (10.0)	7 (13.2)	3 (6.4)	0.328
Pre-XDR with FQ resistance	13 (13.0)	9 (17.0)	4 (8.5)	0.209
XDR	9 (9.0)	5 (9.4)	4 (8.5)	> 0.999
First institution visit, year				0.282
2010	12 (12.0)	5 (9.4)	7 (14.9)	
2011	7 (7.0)	2 (3.8)	5 (10.6)	
2012	13 (13.0)	6 (11.3)	7 (14.9)	
2013	15 (15.0)	6 (11.3)	9 (19.1)	
2014	6 (6.0)	4 (7.5)	2 (4.3)	
2015	17 (17.0)	10 (18.9)	7 (14.9)	
2016	16 (16.0)	12 (22.6)	4 (8.5)	
2017	10 (10.0)	7 (13.2)	3 (6.4)	
2018	4 (4.0)	1 (1.9)	3 (6.4)	

Data are presented as the median [interquartile range] for continuous variables and as the number (percentage) for categorical variables.

AFB, acid fast bacilli; BMI, body mass index; CT, computed tomography; FQ, fluoroquinolone; HIV, human immunodeficiency virus; MDR, multidrug-resistant; SLID, second-line injectable drug; TB, tuberculosis; XDR, extensively drug-resistant.

*Comparisons between Group A and B.

^†^Result from the first visited institution.

^‡^Multidrug-resistant tuberculosis without additional resistance to a fluoroquinolone or a second-line injectable drug.

### Diagnostic modality and treatment before MDR-TB diagnosis

Table 2 lists the diagnostic modality and treatment before MDR-TB diagnosis in Group A and B. LPA for INH and RIF, the Xpert MTB/RIF assay, and bronchoscopy were performed in more patients in Group B than in Group A. Use of first-line anti-TB drugs before MDR-TB diagnosis did not differ between the groups. However, the duration of use was longer in Group A than in Group B, although this was not statistically significant (*P* = 0.061).

**Table 2:** Diagnostic modality, treatment, and time to appropriate treatment initiation and sputum conversion.

	Total (n = 100)	Group A (n = 53)	Group B (n = 47)	*P* Value*
Diagnostic modality, performed				
Line probe assay for isoniazid and rifampin	38 (38.0)	11 (20.8)	27 (57.4)	< 0.001
Xpert MTB/RIF assay	31 (31.0)	9 (17.0)	22 (46.8)	0.001
Bronchoscopy	30 (30.0)	8 (15.1)	22 (46.8)	0.001
Use of first-line anti-TB drugs	83 (83.0)	47 (88.7)	36 (76.6)	0.108
Duration of first-line anti-TB drugs use, days	84.0 [43.0–106.8]	89.5 [46.8–117.0]	70.0 [40.5–97.0]	0.061
Time from first institution visit to initiation of appropriate treatment, days				
Total	93.5 [57.3–127.8]	102.0 [76.0–141.5]	77.0 [18.0–109.0]	0.002
Uncomplicated MDR-TB^†^	87.5 [29.0–126.5]	109.0 [56.5–138.0]	68.5 [10.5–104.3]	0.005
Pre-XDR-TB	97.0 [79.0–126.0]	96.0 [79.5–128.8]	106.0 [77.0–126.0]	0.889
XDR-TB	104.0 [83.5–176.0]	167.0 [96.0–221.5]	90.5 [62.0–163.3]	0.253
Time from first institution visit to negative sputum smear conversion, days^‡^	133.0 [43.3–179.8]	161.0 [122.0–202.3]	69.0 [32.0–143.5]	0.001
Time from first institution visit to negative sputum culture conversion, days	117.0 [71.5–162.5]	129.0 [83.5–209.5]	91.0 [62.0–144.0]	0.003

Data are presented as the median [interquartile range] for continuous variables and as the number (percentage) for categorical variables.

MDR, multidrug-resistant; TB, tuberculosis; XDR, extensively drug-resistant.

*Comparisons between Group A and B.

^†^Multidrug-resistant tuberculosis without additional resistance to a fluoroquinolone or a second-line injectable drug.

^‡^n = 58 (34 patients in Group A and 24 in Group B).

### Time to appropriate treatment

The median time from the first institution visit to appropriate MDR-TB treatment was significantly longer in Group A than for Group B (102.0 *vs*. 77.0 days, respectively; *P* = 0.002; Table 2). Based on resistance levels, the difference between the two groups was maintained for patients with uncomplicated MDR-TB (median of 109.0 *vs*. 68.5 days, respectively; *P* = 0.005). However, there was no difference between the groups in patients with pre-XDR- or XDR-TB (Table 2).

**Fig 1:**
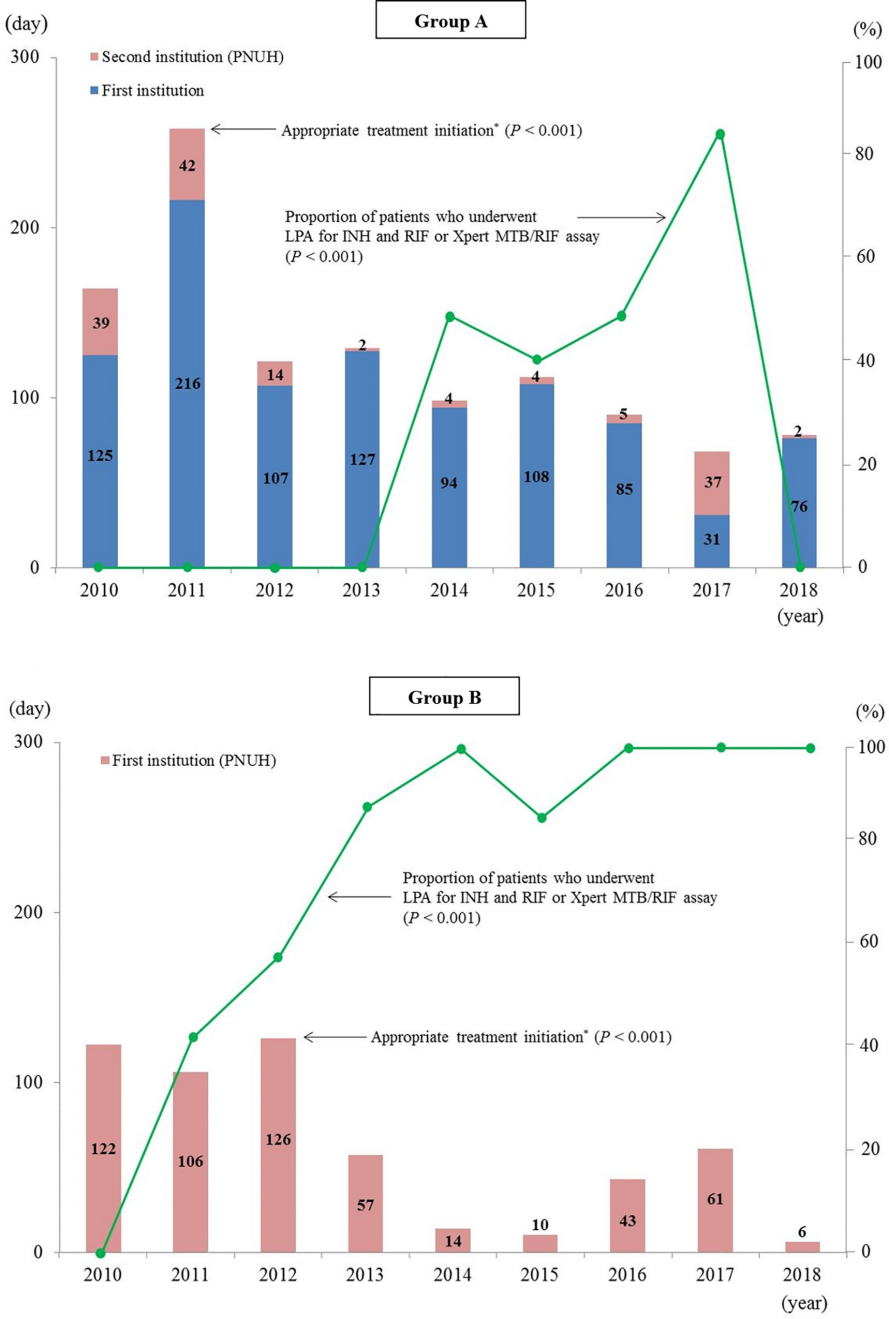
Annual trends in the time from the first institution visit to initiation of appropriate treatment, and the proportion of patients who underwent line probe assays or Xpert MTB/RIF assays. INH, isoniazid; LPA, line probe assay; PNUH, Pusan National University Hospital; RIF, rifampin. *Data are presented as the median.

Fig 1 shows the annual trend in time to appropriate treatment and the proportion of patients in whom LPAs for INH and RIF or Xpert MTB/RIF assays were performed during the study period. The time to appropriate treatment decreased during the study period for both Group A and B. Although the proportion of patients in whom LPAs for INH and RIF or Xpert MTB/RIF assays were performed increased over time in both groups, the proportion itself was lower in Group A than in Group B (34.0 *vs*. 66.0% for the overall study period, respectively; *P* = 0.001).

**Fig 2:**
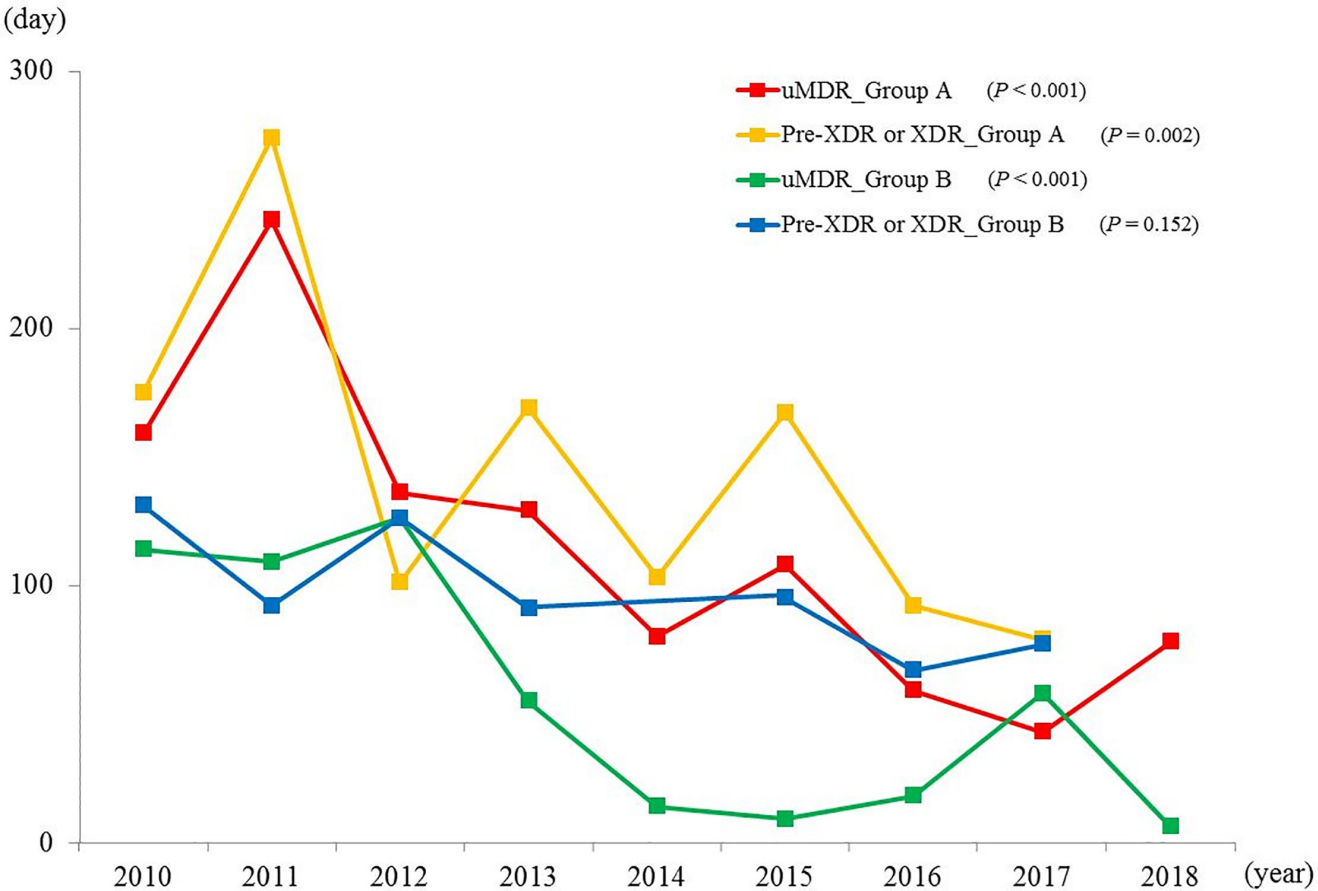
Annual trends in time from first institution visit to initiation of appropriate treatment according to resistance level and group. Data are presented as the median. uMDR, uncomplicated multidrug-resistant; XDR, extensively drug-resistant.

Fig 2 shows the annual trend in time to appropriate treatment according to resistance level and group. The time to appropriate treatment of uncomplicated MDR-TB decreased in both Group A and B during the study period. In patients with pre-XDR- or XDR-TB, the decrease was evident in Group A only.

### Factors affecting late initiation of treatment

A total of 26 patients (26.0%) began MDR-TB treatment < 60 days from the first institution visit. Conversely, 74 patients (74.0%) began MDR-TB treatment ≥ 60 days from the first visit. Logistic regression analyses were performed to investigate factors associated with late initiation of appropriate treatment (≥ 60 days from the first institution visit). Multivariate analyses identified performance of LPAs for INH and RIF, use of the Xpert MTB/RIF assay, and uncomplicated MDR-TB were protective factors against delays in initiation of appropriate treatment (Table 3).

**Table 3:** Factors affecting late initiation of appropriate treatment (≥ 60 days from the first institution visit).

	Univariate			Multivariate		
	OR	95% CI	*P* Value	OR	95% CI	*P* Value
Male sex	1.154	0.458–2.904	0.761			
Age, year	0.980	0.955–1.005	0.121	0.977	0.938–1.017	0.253
Previous treatment history for TB	0.830	0.334–2.060	0.688			
Bilateral disease on chest CT	0.473	0.191–1.171	0.105	0.240	0.053–1.086	0.064
Cavity on chest CT	0.652	0.251–0.652	0.379			
Sputum AFB smear (+)*	0.820	0.329–2.046	0.671			
LPA for isoniazid and rifampin, performed	0.127	0.046–0.350	< 0.001	0.209	0.052–0.842	0.028
Xpert MTB/RIF assay, performed	0.037	0.011–0.121	< 0.001	0.031	0.007–0.148	< 0.001
Bronchoscopy, performed	1.600	0.569–4.500	0.373			
Uncomplicated MDR-TB^†^	0.122	0.027–0.556	0.007	0.082	0.011–0.625	0.016

AFB, acid fast bacilli; CI, confidence interval; CT, computed tomography; LPA, line probe assay; MDR, multidrug-resistant; OR, odds ratio; TB, tuberculosis.

*Result from the first institution visited.

^†^Multidrug-resistant tuberculosis without additional resistance to a fluoroquinolone or a second-line injectable drug.

### Smear and culture conversion and treatment outcomes

The median times from the first institution visit to a negative sputum smear or culture conversion were significantly lower for Group B than for Group A (161.0 *vs*. 69.0 days, respectively; *P* = 0.001 for smear conversion; 129.0 *vs*. 91.0 days, respectively; *P* = 0.003 for culture conversion; Table 2).

At the time of data collection, 88 patients had completed MDR-TB treatment (46 patients in Group A and 42 in Group B). Treatment success rate did not differ between the groups (82.6% in Group A and 78.6% in Group B, *P* = 0.632). Additionally, there were no differences in other outcome categories between the groups. No significant differences were observed in treatment outcomes between patients who began appropriate treatment < 60 days (n = 21) or ≥ 60 days (n = 67) from the first institution visit. Three patients failed treatment; all three began appropriate treatment ≥ 60 days from the first institution visit.

## Discussion

The present study revealed that the time from the first institution visit to initiation of appropriate MDR-TB treatment was longer for patients who were transferred to PNUH after diagnosis of MDR-TB (Group A) than for patients who were initially diagnosed with TB at PNUH (Group B). Although the time to treatment for all MDR-TB patients in both groups decreased during the study period, the time itself was not acceptable, particularly for Group A. In patients with pre-XDR- or XDR-TB, there was no difference in the time to treatment between Group A and Group B, and there was no evidence of a decreasing time to appropriate treatment in Group B during the study period. Our results highlight several concerns regarding diagnosis and treatment of MDR-TB in South Korea: (1) a lack of rapid molecular DST use in institutions other than tertiary referral hospitals or designated TB care centers; (2) a delay in diagnosis of pre-XDR- and XDR-TB using currently available methods, even in well-equipped institutions, and the need for other DST methods able to rapidly detect FQ and SLID resistance; and (3) the possibility of transmission of this difficult-to-treat pathogen within the community in cases of inappropriate treatment.

Compared with other studies that investigated time to treatment of MDR-TB patients, time to treatment in our cohort was longer than that of other studies [4,11,22–24], which was mainly due to the difference in the rate of performance of rapid molecular DSTs. The utility of rapid molecular DSTs such as LPAs for INH and RIF and the Xpert MTB/RIF assay is well known. These tests can decrease the time to diagnosis and treatment initiation in patients with MDR-TB; furthermore, they may improve treatment outcomes [4,11,22–26]. However, molecular DSTs are not commonly used in South Korea, especially in semi-hospitals and clinics. In our study, only 24.5% of patients in Group A were diagnosed with RR- or MDR-TB by molecular DST; most patients were diagnosed with MDR-TB using time-consuming phenotypic DSTs. Although the proportion of patients in Group A in whom a molecular DST was performed increased during the study period, it remained lower than in Group B.

There are several obstacles to the use of molecular DSTs in South Korea. The first is cost. Despite the National TB program, through which diagnosis and treatment of TB is free of charge in South Korea, National Health Insurance does not cover molecular DSTs in all TB patients. Only selected patients can undergo molecular DST free of charge (e.g., recurrent patients and patients in whom treatment has failed). In our study, 61.0% of patients had no previous history of TB. Another Korean study reported that 57.1% of MDR-TB patients were new cases [27]. Under the current policy in South Korea, therefore, more than half of MDR-TB patients will not have the opportunity for rapid diagnosis and treatment. The National TB Program should expand the indications for molecular DST regardless of treatment history for TB. Fortunately, since November 2018, the Xpert MTB/RIF assay, but not LPAs, can be used in all suspected TB patients free of charge in South Korea. The second issue is a lack of awareness of drug resistance and the importance of rapid molecular DSTs among physicians. In our study, molecular DSTs were not commonly performed at other institutions, even for recurring patients. Educational efforts around molecular DSTs could address this problem, especially those directed towards healthcare providers in semi-hospitals and clinics.

Using currently available DSTs, it is not possible to reduce the time to appropriate treatment in patients with pre-XDR- or XDR-TB in South Korea. The only method that detects FQ and SLID resistance in South Korea is phenotypic DST, which is time-consuming. Late detection of FQ and SLID resistance not only delays time to appropriate treatment, but may also worsen treatment outcomes in patients with pre-XDR- or XDR-TB [28–30]. In a previous Korean study, one-third of MDR-TB patients were resistant to FQ and/or SLID [27]. More diagnostic options are required for these significant populations. Recently, the WHO recommended a second-line LPA (GenoType MTBDR*sl*; Hain Lifescience GmbH, Nehren, Germany) to detect FQ and SLID resistance among patients with confirmed RR- or MDR-TB [31]. Its clinical utility for early detection of pre-XDR- or XDR-TB is well-known in various settings [28,32]. However, to date, second-line LPA is performed only in limited settings (mainly for investigational purposes) in South Korea. Discussions regarding the introduction of second-line LPAs into routine practice in South Korea are urgently needed.

Inappropriate treatment before diagnosis of MDR-TB may cause serious public health problems in terms of disease transmission. In our cohort, the median time from the first institution visit to a negative sputum smear or culture conversion was 133 and 117 days, respectively. This long time period may increase the number of exposed individuals in households and in the community. About two-thirds of MDR-TB patients in this study were new patients. Additionally, a previous Korean study showed that the proportion of new patients among total MDR-TB patients did not decrease over time [27]. This phenomenon may be explained, at least in part, by delayed diagnosis and treatment of MDR-TB and persistent transmission in the community. In our cohort, 83% of MDR-TB patients received first-line anti-TB drugs for about 3 months prior to initiation of MDR-TB treatment. Inappropriate treatment with first-line drugs for MDR-TB patients can prevent timely diagnosis of MDR-TB because the bacillary load may be decreased temporarily, thereby reducing the sensitivity of rapid molecular DSTs.

Widespread use of rapid molecular DSTs is not sufficient for rapid diagnosis and treatment of MDR-TB. Several operational problems in various stages of the healthcare system may result in delays in diagnosis and treatment (e.g., delays in sample transportation, the time required for performance of laboratory-based diagnostics, and barriers to efficient patient/physician communication) [4]. Advanced coordination across different levels of the healthcare system is required [6]. Strict monitoring and tracking of the whole course from diagnosis to treatment of TB patients through the National TB Program (e.g., Public-Private Mix collaborations) may help reduce treatment delay in MDR-TB patients [33].

This study has several limitations. First, it is inherently limited due to its retrospective, observational approach. Second, the study was conducted in a single institution with a small number of patients. Therefore, the results may not represent the overall situation in South Korea. Third, information on comorbidities and socioeconomic factors (e.g., level of education, degree of employment, economic status, or accessibility to healthcare facilities) were not fully investigated. These factors may have affected treatment delay or treatment outcome of MDR-TB patients.

In conclusion, the time to appropriate treatment in patients with MDR-TB in South Korea is not acceptable, especially for patients who initially visited institutions other than tertiary referral hospitals or designated TB care centers, and for patients with pre-XDR- or XDR-TB. To reduce the delay in diagnosis and treatment of MDR-TB in South Korea, rapid molecular DSTs should be applied in various healthcare settings and second-line LPAs should be introduced.

## Supporting information

S1 File Raw data of study participants. (SAV)

S2 File Annual report on the notified tuberculosis in Korea: 2017. (PDF)

## References

[1] Dheda K, Gumbo T, Maartens G, Dooley KE, McNerney R, Murray M, et al The epidemiology, pathogenesis, transmission, diagnosis, and management of multidrug-resistant, extensively drug-resistant, and incurable tuberculosis. Lancet Respir Med 2017 pii: S2213–2600(17)30079-6. 10.1016/S2213-2600(17)30079-6 28344011

[2] World Health Organization. Global tuberculosis report 2018. WHO/CDS/TB/2018.20. Geneva, Switzerland: WHO, 2018.

[3] Matteelli A, Migliori GB, Cirillo D, Centis R, Girard E, Raviglion M. Multidrug-resistant and extensively drug-resistant Mycobacterium tuberculosis: epidemiology and control. Expert Rev Anti Infect Ther 2007;5:857–71. 10.1586/14787210.5.5.857 17914919

[4] Jacobson KR, Theron D, Kendall EA, Franke MF, Barnard M, van Helden PD, et al Implementation of genotype MTBDRplus reduces time to multidrug-resistant tuberculosis therapy initiation in South Africa. Clin Infect Dis 2013;56:503–8. 10.1093/cid/cis920 23090928 PMC3552527

[5] Nathanson E, Nunn P, Uplekar M, Floyd K, Jaramillo E, Lonnroth K, et al MDR tuberculosis—critical steps for prevention and control. N Engl J Med 2010;363:1050–8. 10.1056/NEJMra0908076 20825317

[6] Yagui M, Perales MT, Asencios L, Vergara L, Suarez C, Yale G, et al Timely diagnosis of MDR-TB under program conditions: is rapid drug susceptibility testing sufficient? Int J Tuberc Lung Dis 2006;10:838–43. 16898366 PMC8324020

[7] Mukherjee JS, Rich ML, Socci AR, Joseph JK, Virú FA, Shin SS, et al Programmes and principles in treatment of multidrug-resistant tuberculosis. Lancet 2004;363:474–81. 10.1016/S0140-6736(04)15496-2 14962530

[8] Cegielski JP, Kurbatova E, van der Walt M, Brand J, Ershova J, Tupasi T, et al Multidrug-resistant tuberculosis treatment outcomes in relation to treatment and initial versus acquired second-line drug resistance. Clin Infect Dis 2016;62:418–30. 10.1093/cid/civ910 26508515 PMC4725381

[9] Virenfeldt J, Rudolf F, Camara C, Furtado A, Gomes V, Aaby P, et al Treatment delay affects clinical severity of tuberculosis: a longitudinal cohort study. BMJ Open 2014;4:e004818 10.1136/bmjopen-2014-004818 24916087 PMC4067883

[10] Chen Y, Yuan Z, Shen X, Wu J, Wu Z, Xu B. Time to multidrug-resistant tuberculosis treatment initiation in association with treatment outcomes in Shanghai, China. Antimicrob Agents Chemother 2018;62 pii: e02259–17. 10.1128/AAC.02259-17 29437632 PMC5913938

[11] Kipiani M, Mirtskhulava V, Tukvadze N, Magee M, Blumberg HM, Kempker RR. Significant clinical impact of a rapid molecular diagnostic test (Genotype MTBDRplus assay) to detect multidrug-resistant tuberculosis. Clin Infect Dis 2014;59:1559–66. 10.1093/cid/ciu631 25091301 PMC4357804

[12] Dheda K, Gumbo T, Gandhi NR, Murray M, Theron G, Udwadia Z, et al Global control of tuberculosis: from extensively drug-resistant to untreatable tuberculosis. Lancet Respir Med 2014;2:321–38. 10.1016/S2213-2600(14)70031-1 24717628 PMC5526327

[13] Yang C, Luo T, Shen X, Wu J, Gan M, Xu P, et al Transmission of multidrug-resistant Mycobacterium tuberculosis in Shanghai, China: a retrospective observational study using whole-genome sequencing and epidemiological investigation. Lancet Infect Dis 2017;17:275–84. 10.1016/S1473-3099(16)30418-2 27919643 PMC5330813

[14] Shah NS, Yuen CM, Heo M, Tolman AW, Becerra MC. Yield of contact investigations in households of patients with drug-resistant tuberculosis: systematic review and meta-analysis. Clin Infect Dis 2014;58:381–91. 10.1093/cid/cit643 24065336 PMC3890332

[15] Velásquez GE, Yagui M, Cegielski JP, Asencios L, Bayona J, Bonilla C, et al Targeted drug-resistance testing strategy for multidrug-resistant tuberculosis detection, Lima, Peru, 2005–2008. Emerg Infect Dis 2011;17:432–40. 10.3201/eid1703.101553 21392434 PMC3166030

[16] Joh JS, Lee CH, Lee JE, Park YK, Bai GH, Kim EC, et al The interval between initiation of anti-tuberculosis treatment in patients with culture-positive pulmonary tuberculosis and receipt of drug-susceptibility test results. J Korean Med Sci 2007;22:26–9. 10.3346/jkms.2007.22.1.26 17297247 PMC2693564

[17] Korea Centers for Disease Control and Prevention. Annual Report on the Notified Tuberculosis Patients in Korea: 2017. http://tbzero.cdc.go.kr/tbzero/board/boardView.do?leftMenuId=48&paramMenuId=77&boardSeq=4824&crudType=R. Accessed 1 Mar 2018.

[18] World Health Organization. Definitions and reporting framework for tuberculosis-2013 revision. WHO/HTM/TB/2013.2. Geneva, Switzerland: WHO, 2013.

[19] World Health Organization. Guidelines for the programmatic management of drug-resistant tuberculosis—2011 update. WHO/HTM/TB/2011.6. Geneva, Switzerland: WHO, 2011. 23844450

[20] World Health Organization. Companion handbook to the WHO guidelines for the programmatic management of drug-resistant tuberculosis. WHO/HTM/TB/2014.11. Geneva, Switzerland: WHO, 2014. 25320836

[21] World Health Organization. WHO treatment guidelines for drug-resistant tuberculosis—2016 update. WHO/HTM/TB/2016.04. Geneva, Switzerland: WHO, 2016. 27748093

[22] Singla N, Satyanarayana S, Sachdeva KS, Van den Bergh R, Reid T, Tayler-Smith K, et al Impact of introducing the line probe assay on time to treatment initiation of MDR-TB in Delhi, India. PLoS One 2014;9:e102989 10.1371/journal.pone.0102989 25058124 PMC4109962

[23] Eliseev P, Balantcev G, Nikishova E, Gaida A, Bogdanova E, Enarson D, et al The impact of a line probe assay based diagnostic algorithm on time to treatment initiation and treatment outcomes for multidrug resistant TB patients in Arkhangelsk region, Russia. PLoS One 2016;11:e0152761 10.1371/journal.pone.0152761 27055269 PMC4824472

[24] Stagg HR, White PJ, Riekstiņa V, Cīrule A, Šķenders Ģ, Leimane V, et al Decreased time to treatment initiation for multidrug-resistant tuberculosis patients after use of Xpert MTB/RIF test, Latvia. Emerg Infect Dis 2016;22:482–90. 10.3201/eid2203.151227 26889608 PMC4766893

[25] Iruedo J, O'Mahony D, Mabunda S, Wright G, Cawe B. The effect of the Xpert MTB/RIF test on the time to MDR-TB treatment initiation in a rural setting: a cohort study in South Africa's Eastern Cape Province. BMC Infect Dis 2017;17:91 10.1186/s12879-017-2200-8 28109255 PMC5251218

[26] Steingart KR, Schiller I, Horne DJ, Pai M, Boehme CC, Dendukuri N. Xpert® MTB/RIF assay for pulmonary tuberculosis and rifampicin resistance in adults. Cochrane Database Syst Rev 2014:CD009593 10.1002/14651858.CD009593.pub3 24448973 PMC4470349

[27] Mok JH, Kang BH, Lee T, Lee HK, Jang HJ, Cho YJ, et al Additional drug resistance patterns among multidrug-resistant tuberculosis patients in Korea: implications for regimen design. J Korean Med Sci 2017;32:636–41. 10.3346/jkms.2017.32.4.636 28244290 PMC5334162

[28] Lee YS, Lee BY, Jo KW, Shim TS. Performance of the GenoType MTBDRsl assay for the detection second-line anti-tuberculosis drug resistance. J Infect Chemother 2017;23:820–5. 10.1016/j.jiac.2017.08.010 29066216

[29] Olaru ID, von Groote-Bidlingmaier F, Heyckendorf J, Yew WW, Lange C, Chang KC. Novel drugs against tuberculosis: a clinician's perspective. Eur Respir J 2015;45:1119e31. 25431273 10.1183/09031936.00162314

[30] Falzon D, Schunemann HJ, Harausz E, Gonzalez-Angulo L, Lienhardt C, Jaramillo E, et al World Health Organization treatment guidelines for drugresistant tuberculosis, 2016 update. Eur Respir J 2017;49 pii: 1602308 10.1183/13993003.02308-2016 28331043 PMC5399349

[31] World Health Organization. The use of molecular line probe assays for the detection of resistance to second-line anti-tuberculosis drugs Policy guidance. WHO/HTM/TB/2016.07. Geneva, Switzerland: WHO, 2016.

[32] Theron G, Peter J, Richardson M, Warren R, Dheda K, Steingart KR. GenoType® MTBDRsl assay for resistance to second-line anti-tuberculosis drugs. Cochrane Database Syst Rev 2016;9:CD010705 10.1002/14651858.CD010705.pub3 27605387 PMC5034505

[33] Kim JH, Yim JJ. Achievements in and challenges of tuberculosis control in South Korea. Emerg Infect Dis 2015;21:1913–20. 10.3201/eid2111.141894 26485188 PMC4622233

