# Supplementary material for: Time to appropriate treatment in patients with multidrug-resistant tuberculosis in South Korea: Are we still in 2010?
Source: PLoS One. 2019 Apr 25;14(4):e0216084. doi: 10.1371/journal.pone.0216084 (PMC6483266; doi:10.1371/journal.pone.0216084)
Supplement: S2 File — (PDF) [file pone.0216084.s002.pdf]

11-1351159-000020-10

Annual Report on the Notified Tuberculosis in Korea

2017

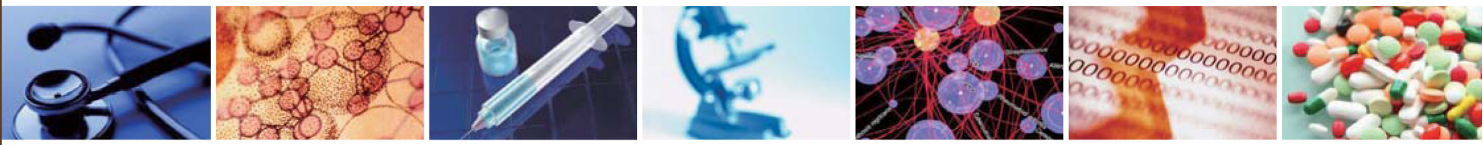

Centers for Disease Control & Prevention

Annual Report on the Notified Tuberculosis in Korea, 2017

Published by
Korea Centers for Disease Control & Prevention

Publisher Jung Eunkyeong
(Director of Korea Centers for Disease Control & Prevention)

Publish committee
(Director of Center for Disease Prevention, Korea Centers for Disease Control & Prevention)
Go Unyeong
(Division of TB & HIV control, Korea Centers for Disease Control & Prevention)
Shin Insik
Kim Jonghee
Cha Jeongok
Choi Junkil
Park Yongjoon
Park Wonseo
Jeong Hyeran
Cho Sangsik
Jang Sungryul

Published on July 2018

Address (28159) Osong Health technology Administration Complex, 187, Osongsaengmyeong 2-ro, Osong-eup, Heungdeok-gu, Cheongju-si, Chungcheongbuk-do, Korea Center for Disease Control and Prevention

Telephone (043) 719-7341

Fax (043) 719-7339

Number of statistics approved by KNSO : 92007

Contents

Ⅰ. Introduction ········· 1

1. Purpose ········· 2
2. Applicable law ········· 2
3. History and announcements of results ········· 2
4. Tuberculosis case notifications ········· 6
5. Definitions ········· 6
6. Analysis method ········· 9
7. Notes ········· 11

Ⅱ. Tuberculosis case notifications, 2001-2017 ········· 13

[Table 1] (New) TB notification cases and rates, 2001-2017 ········· 14
[Table 2] TB notification cases by treatment history, 2001-2017 ········· 15
[Figure 1] (New) TB notification rates, 2001-2017 ········· 16
[Figure 2] Age-standardized (new) TB notification rates, 2001-2017 ········· 16

Ⅲ. Tuberculosis case notifications, 2017 ········· 13

[Table 3] (New) TB notification cases and rates by province, gender and age, 2017 ········· 18
[Table 4] (New) TB notification cases by regions, 2017 ········· 30
[Figure 3] (New) TB notification rates by gender and age, 2017 ········· 34
[Figure 4] (New) TB notification rates by province, 2017 ········· 34

Ⅳ. New Tuberculosis case notifications, 2001-2017 ········· 35

[Table 5] New TB notification cases and rates by gender and age, 2001-2017 ········· 36
[Table 6] New TB notification cases and rates by regions, 2005-2017 ········· 42
[Table 7] New TB notification cases and rates by pathological location and result of sputum smear test, 2005-2017 ········· 44
[Table 8] New TB notification cases and rates by type of healthcare provider, 2001-2017 · 45
[Table 9] New TB notification cases in foreign born, 2001-2017 ········· 46

Ⅴ. Multidrug-resistant tuberculosis case notifications, 2011-2017 ········· 47

[Table 10] Multidrug-resistant TB notification cases, 2011-2017 ········· 48
[Table 11] Extensively drug-resistant TB notification cases, 2011-2017 ········· 50

Ⅵ. APPENDIX ········· 53

1. The number of deaths of tuberculosis by gender and age, 2001-2016 ········· 54
2. Tuberculosis Case Notification · Reporting Form ········· 58

Ⅰ. Introduction

1. Purpose

The annual report was written to analyze information of tuberculosis patient who was diagnosed or treated at the national public health centers and clinics collected through the integrated disease and health control system and to plan, implement and evaluate the national TB control policy based on the results.

2. Applicable law

Act on the prevention and management of infectious diseases, Article 11(Notification by doctors) and Article 12(Notification by other reporters) and Act on TB prevention, Article 8(Notification obligations of health care providers).

3. History and announcements of results

A. History

- ○ 1976 Prepared the nationally approved statistics (Statistics approval No.: 11756, Current situation of nationwide tuberculosis patients)
- ○ 2000 Constructed the Korean Tuberculosis Surveillance System (KTBS).
- ○ 2002 Published the "Annual Report on the Notified Tuberculosis in Korea" for the first time.
  - It is prepared based on the data on the tuberculosis patients reported by nationwide public health clinics and private clinics and hospitals.
- ○ 2005 Constructed the Tubercular Bacillus Examination Management Information System (TBIS).
- ○ 2006 Constructed the National Tuberculosis Management Image Information System (TB-PACS).

○ 2008 The KTBS is transferred from The Korean National Tuberculosis Association to Korea Centers for Disease Control and Prevention.

○ 2008 Changed the period for preparation of nationally approved statistic to every year.

○ 2009 Constructed the Tuberculosis Information Management System (TBNET).

- The filling and reporting of tuberculosis patients and the examination work in the public health clinic are integrated into the system.

○ 2011 The tuberculosis work system inside the integral disease and health control system is integrated.

- The TBNET under operation inside the Korea Centers for Disease Control and Prevention is integrated into the integral disease and health control system.

○ 2013 Changed the statistical approval No. for the nationally approved statistics (No. 92007, Current situation of tuberculosis)

B. Public announcement of the results

○ Provided the current situation of tuberculosis through the Korea Statistical Information Service of the Korea Statistics Service (http://kosis.kr)

○ Provided the periodical files through the website.

(1) Website of Korea Centers for Disease Control and Prevention (http://www.cdc.go.kr)

(2) Website of Tuberculosis ZERO (http://tbzero.cdc.go.kr)

4. Tuberculosis case notifications

A. Notifications subjects

- The director of medical institution, physicians and other medical personnel: They have to notify to the head of public health center.
- Commander : The military medical team in Army, Navy, Air Force and other units under the Ministry of Defense should report it to their commander. Then, commander ought to notify to the head of competent public health center.
- Other reporters : In the following cases, the reporters should ask for physician's diagnosis or examination. They should notify to the head of competent public health center.
  - The representative of a household or family members in general household
  - A manager, director or head of school, hospital, public office, company, theater, church, public transportation such as ship, air plane and train, variable office, business office, restaurant, hotel and place where many people can come together in.

- Report by the head of public health center: The head of public health center who was notified by the reporters should report to the governor or mayor of special self-governing province. The city mayor, governor and the head of district ought to make a report to the Minister of Health and Welfare or the governor chief respectively.

B. Notification period: immediate in the following cases

○ Notification

(1) When a physician diagnosed or treated a TB patient or suspected TB patient.

(2) When a TB patient or suspected TB patient expired or a physician conducted a postmortem examination on a TB patient's body.

○ Reporting: Result of treatment of TB patient or suspected TB patient notified according to the notification (1) above.

C. Target for notification : TB patient and TB suspected patient

○ TB patient : An individual, who is infected by a TB pathogen, has clinical symptoms of TB, and shows positive result in Mycobacterium tuberculosis test.

○ Suspected TB patient : An individual, who is found to have TB infection in terms of clinical, radiological and histological indications but is not confirmed to be positive result in Mycobacterium tuberculosis test.

- Diagnostic criteria for notification
  - Patient : An individual who shows the clinical symptoms of TB and is confirmed to have been infected by a clinical pathogen in the inspection criteria for diagnosis.
  - Suspected patient : An individual, who has the TB infection in terms of clinical, radiological and histological indications but does not have a bacteriological test result in inspection criteria for diagnosis.
- Inspection criteria for diagnosis
  - Smear–positive in test from a patient specimen (sputum, bronchial washing, body fluids, tissues)
  - Culture–positive in test for Mycobacterium tuberculosis with a patient specimen (sputum, bronchial washing, body fluids, tissues)
    * Especially, *M. bovis* should be identified in the culture for confirmation of diagnosis.
  - Unusual genes detect in patient specimen (sputum, bronchial washing, body fluids, tissues)

[Diagnostic criteria of infectious disease
(Report No. 2017–4, of the Korea Centers for Disease Control and Prevention, July. 18, 2017)]

D. Methods of Notification

○ Where to notify : The head of public health center

○ Method of notification: Fax and website (http://is.cdc.go.kr Integrated disease and health control system – TB control)

○ Notification form: Notification and report form for TB patients and others (Accompanying paper format according to the Enforcement Regulation of Tuberculosis Prevention Act)

5. Definitions

A. Tuberculosis patient classification

1) Classification by treatment history[1)]

Classification	Definition
New patient	A patient who has never been treated for Tuberculosis<br>※ who has taken anti-tuberculosis drugs for less than 1 month<br>※ who has received treatment in a hospital as a new case but has been transferred to other hospital without cure/completion/failure/default
Previously treated patient	A patient who has received 1 month or more of anti-TB drugs in past<br>※ They can be classified further by the outcome of their most recent course of treatment as follows
Relapse	A patient who has previously been treated for TB, were declared cured or treatment completed at the end of their most recent course of treatment, and are now diagnosed with a recurrent episode of TB
Treatment after failure	A patient who has previously been treated for TB and whose treatment failed at the end of their most recent course of treatment
Treatment after loss to follow-up	A patient who has previously been treated for TB and were declared lost to follow-up at end of their most recent course of treatment
Other preciously treated	A patient who has previously been treated for TB but whose outcome after their most recent course of treatment is unknown or undocumented.
Patient with unknown previous TB treatment history	A patient who has unknown previous TB treatment history

1) According to the revision of Enforcement Regulation of Tuberculosis Prevention Act (July 29, 2014), the classifications of TB patients into 'new patient' and 'previously treated patient (relapse, treatment after failure, treatment after default, transferred-in, chronic) and other' were changed to those of 'new patient', 'previously treatment patient(relapse, treatment after failure, treatment after loss to follow-up and other previously treated') and 'patient with unknown previous TB treatment history'.

2) Classification by pathological location

Classification	Contents	International Statistical Classification of Diseases (7th Korean Standard Classification of Diseases, KCD)
Pulmonary tuberculosis	Tuberculosis, or miliary tuberculosis which invades into Lung parenchyma or bronchus and tracheobronchial tree	A15.0 Tuberculosis confirmed by sputum microscopy with or without culture<br>A15.1 Tuberculosis of lung without cavitation or unspecified, confirmed by sputum microscopy with or without culture<br>A15.2 Tuberculosis confirmed histologically<br>A15.3 Tuberculosis confirmed by unspecified means<br>A15.5 Tuberculosis of larynx, trachea and bronchus, confirmed bacteriologically and histologically<br>A16.0 Tuberculosis of lung, bacteriologically and histologically negative<br>A16.1 Tuberculous of lung with cavitation, bacteriological and histological examination not done<br>A16.2 Tuberculosis of lung, without mention of bacteriological or histological confirmation<br>A16.4 Tuberculosis of larynx, trachea and bronchus, without mention of bacteriological or histological confirmation<br>A19 Miliary tuberculosis
Extra pulmonary tuberculosis	Tuberculous pleurisy, intrathoracic lymph node tuberculosis, tuberculosis of the nervous system, bone and joint tuberculosis, tuberculosis of the urogenital system, tuberculosis of intestine and peritoneum, and mesenteric lymph nodes	A15.4 Tuberculosis of intrathoracic lymph nodes, confirmed bacteriologically and histologically<br>A15.6 Tuberculous pleurisy, confirmed bacteriologically and histologically<br>A15.7 Primary respiratory tuberculosis, confirmed bacteriologically and histologically<br>A15.8 Other respiratory tuberculosis, confirmed bacteriologically and histologically<br>A15.9 Respiratory tuberculosis, confirmed bacteriologically and histologically<br>A16.3 Tuberculosis of intrathoracic lymph nodes, without mention of bacteriological or histological confirmation<br>A16.5 Tuberculous pleurisy, without mention of bacteriological or histological confirmation<br>A16.7 Primary respiratory tuberculosis without mention of bacteriological or histological confirmation<br>A16.8 Other respiratory tuberculosis, without mention of bacteriological or histological confirmation<br>A16.9 Respiratory tuberculosis unspecified, without mention of bacteriological or histological confirmation<br>A17 Tuberculosis of nervous system<br>A18 Tuberculosis of other organs

* Tuberculosis of larynx, trachea and bronchus is included in pulmonary tuberculosis since 2012 annual report.

[Reference] 1. Definitions and reporting framework for tuberculosis-2013 revision, WHO, 2013.
2. Global Tuberculosis Control, WHO report, WHO, 2001.

3) Classification by result of sputum smear examination

Classification	Contents
Smear positive	Smear positive for AFB of the first tuberculosis examination

4) Classification by drug-resistant tuberculosis

Classification	Contents
Multidrug -resistant tuberculosis	(Multidrug-resistant TB, MDR-TB) : U84.30<br>TB which is caused by tubercle bacilus which is resistant to more than two antituberculosis drugs including Isoniazid and Rifampin
Extensively drug -resistant tuberculosis	(Extensively drug-resistant TB, XDR-TB) : U84.31<br>TB which shows the resistance to both of Isoniazid and Rifampin, more than one of Quinolones and more than one of 3 injections (capreomycin, kanamycin and amikacin)

* According to the 7th revision of Korean Standard Classification of Diseases (KCD) (effective on Jan. 1, 2016), the revised multidrug-resistant tuberculosis code and the extensively drug-resistant tuberculosis code will take effect on Jan.1, 2016. (Multidrug-resistant TB : U88.0 → U84.30, Extensively drug-resistant TB : U88.1 → U84.31)

B. (New) Tuberculosis notification rate

1) (New) TB notification rates

It can be obtained by dividing the total number of notified (new) TB cases by a mid-year population to show in a way that the number of patient per every 100,000.

- The case notification rates uses the mid-year population of the year as standard population.

(Source: Korean statistical information service(http://kosis.kr), Statistics Korea)

$$\frac{\text{Total number of (new) TB notification case}}{\text{Mid-year population of the year}} \times 100{,}000$$

2) Age-standardized (new) TB notification rate

The age-standardized (new) TB notification rate is obtained by applying the (new) TB notification rate by age to standard population by age in order to prevent it from affected by the difference in age group.

- Standard population (5 years period) adopted the national population of 2005.

(Source : annual report on the causes of death statistics of 2016, Statistics Korea)

$$\frac{\Sigma(\text{(New) TB notification rate by age} \times \text{Standard population by age})}{\text{Standard population}}$$

6. Analysis method

A. Confirmed subjects for analysis

○ Raw data : Notified cases of tuberculosis (suspected) from January 1, 2017 to December 31, 2017. As of February 27, 2018, 51,019 cases.

○ Nontuberculosis cases are excluded.

B. Case classification

○ Classification by treatment history.

- In the case of duplicate notifications, it is classified by the order of 'new patient', 'previously treated patient(relapse, treatment after failure, treatment after loss to follow-up and other previously treated') and 'patient with unknown previous TB treatment history'.
- Among the new patients notified this year, those who have been notified before or whose notification has been delayed for more than 1 year are classified as 'other'.

○ Classification by result of examination and pathological location

- If the result is positive in Tuberculosis first examination of sputum smear, it will be classified smear-positive.
  - The duplicated cases will be reclassified by the priority order of positive, negative, unknown and non examination.
- Classification of pulmonary TB and extra pulmonary TB follow the Disease Code.
  - Duplicated TB cases give priority to pulmonary TB.

○ Multidrug-resistant and extensively drug-resistant tuberculosis

- A patient who is reported to have multidrug-resistant tuberculosis in the year or is converted to multidrug-resistant TB among past cases.
- Patient with multidrug-resistant tuberculosis in the year = $A \cup B - C$

- A : A patient who is reported to have multidrug-resistant TB among the patient notified in 2017
- B : A patient who was found to be multidrug-resistant in the test results found in drug-resistant examination data between 2016 and 2017
- C : A patient who was reported to have multidrug-resistant TB in the past.

- The same analysis methods applies to the extensively drug-resistant TB as to multidrug-resistant TB.

※ Patients with multidrug resistance include those who have the extensively drug resistance(However, the patients who were filed as those who had the multidrug resistance in the past and were also filed as having the extensively drug resistance in the relevant year were included in the category of patients who have the extensively drug resistance in the relevant year only.)

7. Notes

A. This annual report was made based on the TB cases notified from public health centers, hospitals and medical centers for a year (The National Approval Statistic, Statistics Korea). So, there may be difference between notified new case notification rate and exact TB incidence rate.

B. Number of population for calculation of new case notification rate is based on the mid-year population of the year made by the Statistics Korea. Therefore, there is difference between this report and previous reports based on the estimated population up to year 2010.

C. Age-standardized (new) TB notification rates (Figure 2) used the standard population of 2005(5 years period) which is used as standard population in the death statistics of Statistics Korea in order to compensate the difference in population in age group.

D. Case notification region (province and district) of the annual report is based on the location of the patient. (TB patients without residental address are counted as the resident of the reported region.)

E. All of statistics include foreigners. (The statistics on foreigners will be given in separate sheet.)

F. The number of deaths of tuberculosis by gender and age in this annual report is sourced from “Annual Report on the Causes of Death Statistics” released on the end of every September by Statistics Korea. (The number of deaths of tuberculosis in 2017 is acquired from the report released by Statistics Korea at the end of September 2018.)

Ⅱ. Tuberculosis case notifications, 2001-2017

[Table 1] (New) TB notification cases and rates, 2001–2017

(Unit: person (person/100,000 population))

Year	Total cases	New cases
2001	46,082<br>(96.3)	34,123<br>(71.3)
2002	43,040<br>(89.4)	32,010<br>(66.5)
2003	40,500<br>(83.8)	30,687<br>(63.5)
2004	41,735<br>(86.1)	31,503<br>(65.0)
2005	46,969<br>(96.5)	35,269<br>(72.4)
2006	46,284<br>(94.7)	35,361<br>(72.3)
2007	45,597<br>(92.8)	34,710<br>(70.6)
2008	44,174<br>(89.4)	34,157<br>(69.1)
2009	47,302<br>(95.3)	35,845<br>(72.2)
2010	48,101<br>(96.4)	36,305<br>(72.8)
2011	50,491<br>(100.8)	39,557<br>(78.9)
2012	49,532<br>(98.4)	39,545<br>(78.5)
2013	45,292<br>(89.6)	36,089<br>(71.4)
2014	43,088<br>(84.9)	34,869<br>(68.7)
2015	40,847<br>(80.2)	32,181<br>(63.2)
2016	39,245<br>(76.8)	30,892<br>(60.4)
2017	36,044<br>(70.4)	28,161<br>(55.0)

[Table 2] TB notification cases by treatment history, 2001–2017

(Unit: person)

Year	Total cases	New cases	Previously treated cases[1)2)]					Cases with unknown previous TB treatment history	Other[3)]
			Sub-total	Relapse	Treatment after failure	Treatment after loss to follow-up	Other previously treated		
2001	46,082	34,123	11,679	6,742	500	914	-	-	280
2002	43,040	32,010	10,797	6,270	429	772	-	-	233
2003	40,500	30,687	9,647	5,693	359	713	-	-	166
2004	41,735	31,503	9,914	5,691	405	803	-	-	318
2005	46,969	35,269	9,012	5,891	363	711	-	-	2,688
2006	46,284	35,361	8,535	5,558	281	773	-	-	2,388
2007	45,597	34,710	8,299	5,403	314	784	-	-	2,588
2008	44,174	34,157	7,917	5,227	289	734	-	-	2,100
2009	47,302	35,845	8,655	5,624	309	896	-	-	2,802
2010	48,101	36,305	8,794	5,637	267	949	-	-	3,002
2011	50,491	39,557	9,419	5,844	206	1,165	-	-	1,515
2012	49,532	39,545	7,750	5,215	121	836	-	-	2,237
2013	45,292	36,089	8,128	5,490	81	822	-	-	1,075
2014	43,088	34,869	6,254	5,069	98	909	178	252	1,713
2015	40,847	32,181	6,211	4,934	101	829	347	426	2,029
2016	39,245	30,892	6,087	4,981	71	707	328	562	1,704
2017	36,044	28,161	5,637	4,696	49	623	269	610	1,636

1) According to the revision of Enforcement Regulation of Tuberculosis Prevention Act (July 29, 2014), the classifications of TB patients into 'new cases' and 'previously treated cases (relapse, treatment after failure, treatment after default, transferred-in, chronic) and other' were changed to those of 'new cases', 'previously treatment cases(relapse, treatment after failure, treatment after loss to follow-up and other previously treated') and 'cases with unknown previous TB treatment history'.
2) The previous categories of 'transferred-in' and 'chronic' have been deleted. The cases which were classified as the 'transferred-in' and 'chronic' from 2001 to 2013 were included the subtotal of previously treated cases(For the details on the number of 'transferred-in' and 'chronic', see "Annual report on the notified Tuberculosis in korea 2013".
3) Among the new cases notified this year, those who have been notified before or whose notification has been delayed for more than 1 year are classified as 'other'.

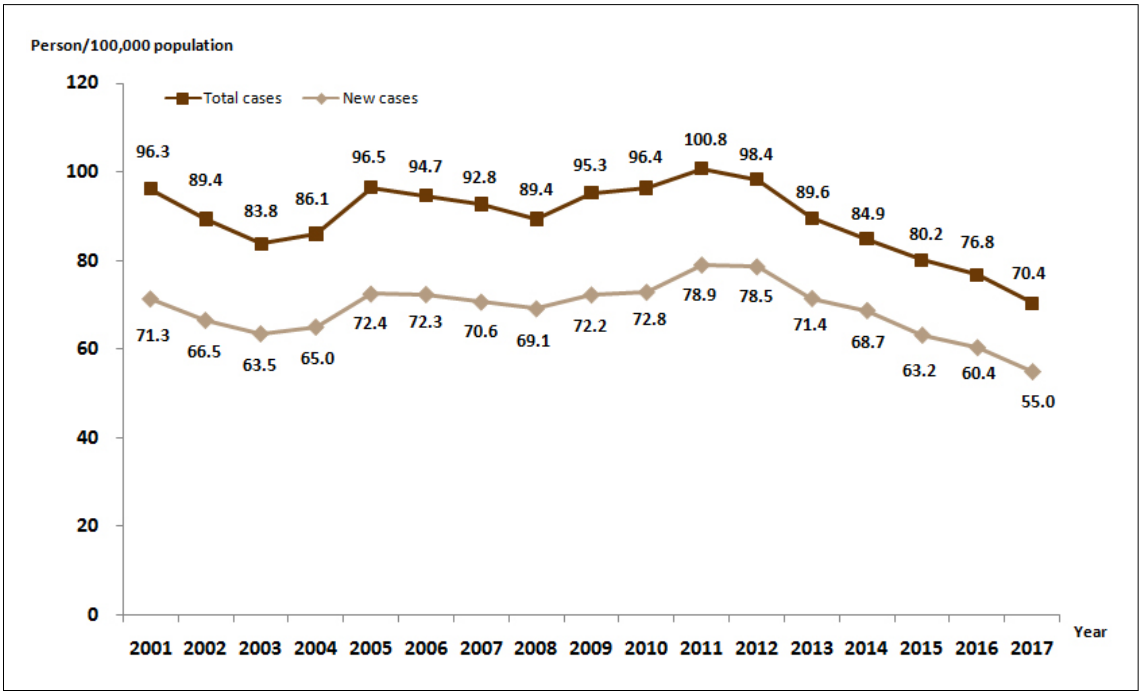

[Figure 1] (New) TB notification rates, 2001-2017

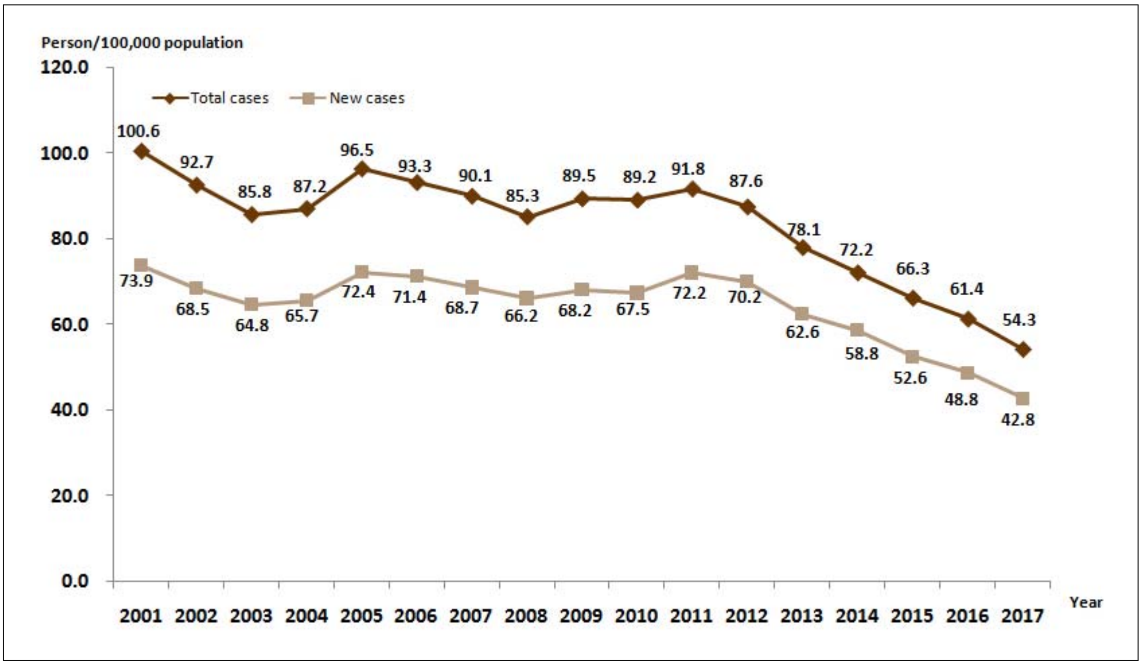

* Age-standardized (new) TB notification rates: The national population in 2005 was used as standard population (Source: the causes of death statistics in 2016, Statistics Korea)

[Figure 2] Age-standardized (new) TB notification rates, 2001-2017

Ⅲ. Tuberculosis case notifications, 2017

[Table 3] (New) TB notification cases and rates by province, gender and age, 2017

Province		Gender \ Age	Total	0–4	5–9	10–14	15–19	20–24	25–29	30–34	35–39
Whole Country	Total cases	Total	36,044	16	8	80	667	1,323	1,676	1,530	1,718
			(70.4)	(0.7)	(0.3)	(3.4)	(22.0)	(37.8)	(51.5)	(45.1)	(43.0)
		Male	21,645	8	5	39	410	691	847	864	971
			(84.6)	(0.7)	(0.4)	(3.2)	(25.9)	(37.3)	(49.7)	(49.6)	(47.7)
		Female	14,399	8	3	41	257	632	829	666	747
			(56.1)	(0.8)	(0.3)	(3.6)	(17.7)	(38.4)	(53.6)	(40.4)	(38.1)
	New cases	Total	28,161	16	8	75	622	1,135	1,429	1,244	1,379
			(55.0)	(0.7)	(0.3)	(3.2)	(20.5)	(32.4)	(43.9)	(36.7)	(34.5)
		Male	16,147	8	5	36	386	581	711	686	759
			(63.1)	(0.7)	(0.4)	(3.0)	(24.4)	(31.4)	(41.7)	(39.4)	(37.3)
		Female	12,014	8	3	39	236	554	718	558	620
			(46.8)	(0.8)	(0.3)	(3.5)	(16.3)	(33.6)	(46.4)	(33.8)	(31.6)
Seoul	Total cases	Total	6,663	4	0	13	155	279	423	356	374
			(68.5)	(1.1)	(0.0)	(3.3)	(29.9)	(41.2)	(55.9)	(46.0)	(46.3)
		Male	4,166	1	0	5	95	125	191	200	198
			(87.4)	(0.5)	(0.0)	(2.5)	(35.5)	(36.8)	(50.9)	(51.7)	(48.9)
		Female	2,497	3	0	8	60	154	232	156	176
			(50.4)	(1.7)	(0.0)	(4.2)	(24.0)	(45.5)	(60.9)	(40.3)	(43.7)
	New cases	Total	5,226	4	0	11	145	239	366	296	311
			(53.7)	(1.1)	(0.0)	(2.8)	(28.0)	(35.3)	(48.4)	(38.3)	(38.5)
		Male	3,120	1	0	4	89	100	163	164	166
			(65.4)	(0.5)	(0.0)	(2.0)	(33.2)	(29.4)	(43.5)	(42.4)	(41.0)
		Female	2,106	3	0	7	56	139	203	132	145
			(42.5)	(1.7)	(0.0)	(3.7)	(22.4)	(41.1)	(53.2)	(34.1)	(36.0)
Busan	Total cases	Total	2,462	1	0	4	47	100	123	92	116
			(71.4)	(0.8)	(0.0)	(3.0)	(25.7)	(42.6)	(56.7)	(43.1)	(45.1)
		Male	1,535	1	0	2	27	57	68	51	57
			(90.4)	(1.5)	(0.0)	(2.9)	(28.1)	(45.7)	(60.0)	(46.6)	(43.4)
		Female	927	0	0	2	20	43	55	41	59
			(53.0)	(0.0)	(0.0)	(3.1)	(23.0)	(39.1)	(53.2)	(39.3)	(46.8)
	New cases	Total	1,861	1	0	3	39	88	99	73	90
			(54.0)	(0.8)	(0.0)	(2.2)	(21.3)	(37.5)	(45.7)	(34.2)	(35.0)
		Male	1,109	1	0	1	23	51	51	42	45
			(65.3)	(1.5)	(0.0)	(1.4)	(23.9)	(40.9)	(45.0)	(38.4)	(34.3)
		Female	752	0	0	2	16	37	48	31	45
			(43.0)	(0.0)	(0.0)	(3.1)	(18.4)	(33.6)	(46.4)	(29.7)	(35.7)

(Unit : person (person/100,000 population)) – continued –

40–44	45–49	50–54	55–59	60–64	65–69	70–74	75–79	80+	unknown
2,033	2,512	2,838	3,523	3,021	2,587	2,847	3,733	5,932	0
(49.5)	(55.4)	(69.1)	(83.4)	(95.1)	(114.4)	(161.2)	(247.8)	(387.2)	–
1,279	1,681	2,025	2,560	2,156	1,725	1,758	2,042	2,584	0
(61.4)	(73.3)	(97.0)	(121.3)	(138.1)	(158.7)	(218.8)	(327.6)	(535.3)	–
754	831	813	963	865	862	1,089	1,691	3,348	0
(37.3)	(37.1)	(40.3)	(45.6)	(53.6)	(73.4)	(113.2)	(191.5)	(319.1)	–
1,602	1,902	2,088	2,623	2,240	1,972	2,196	2,919	4,711	0
(39.0)	(41.9)	(50.9)	(62.1)	(70.5)	(87.2)	(124.4)	(193.8)	(307.5)	–
981	1,210	1,433	1,837	1,534	1,271	1,268	1,507	1,934	0
(47.1)	(52.7)	(68.6)	(87.1)	(98.3)	(116.9)	(157.8)	(241.8)	(400.7)	–
621	692	655	786	706	701	928	1,412	2,777	0
(30.7)	(30.9)	(32.5)	(37.2)	(43.7)	(59.7)	(96.4)	(159.9)	(264.7)	–
407	495	567	706	621	485	472	543	763	0
(52.1)	(58.3)	(76.1)	(90.9)	(100.3)	(109.3)	(137.0)	(209.7)	(322.1)	–
265	328	396	537	459	333	312	343	378	0
(68.3)	(78.2)	(107.6)	(143.8)	(156.0)	(158.8)	(194.8)	(297.0)	(468.4)	–
142	167	171	169	162	152	160	200	385	0
(36.2)	(38.9)	(45.3)	(41.9)	(49.8)	(65.0)	(86.8)	(139.5)	(246.5)	–
330	386	418	517	463	355	362	430	593	0
(42.3)	(45.5)	(56.1)	(66.6)	(74.8)	(80.0)	(105.1)	(166.1)	(250.3)	–
207	241	280	379	328	235	232	262	269	0
(53.3)	(57.4)	(76.1)	(101.5)	(111.5)	(112.1)	(144.8)	(226.9)	(333.3)	–
123	145	138	138	135	120	130	168	324	0
(31.3)	(33.8)	(36.6)	(34.2)	(41.5)	(51.3)	(70.6)	(117.2)	(207.5)	–
128	157	193	275	242	213	201	260	310	0
(50.4)	(54.2)	(69.8)	(86.3)	(91.9)	(110.2)	(143.4)	(236.5)	(317.7)	–
74	105	155	175	189	150	134	154	136	0
(57.8)	(73.6)	(114.8)	(114.6)	(149.4)	(163.6)	(206.4)	(332.4)	(443.4)	–
54	52	38	100	53	63	67	106	174	0
(42.8)	(35.4)	(26.8)	(60.2)	(38.7)	(62.0)	(89.0)	(166.6)	(260.1)	–
98	114	134	200	180	159	150	192	241	0
(38.6)	(39.3)	(48.4)	(62.7)	(68.4)	(82.3)	(107.0)	(174.6)	(247.0)	–
53	74	106	118	138	112	92	104	98	0
(41.4)	(51.9)	(78.5)	(77.3)	(109.1)	(122.2)	(141.7)	(224.5)	(319.5)	–
45	40	28	82	42	47	58	88	143	0
(35.7)	(27.2)	(19.8)	(49.4)	(30.7)	(46.3)	(77.1)	(138.3)	(213.8)	–

[Table 3] (New) TB notification cases and rates by province, gender and age, 2017

Province		Age / Gender	Total	0–4	5–9	10–14	15–19	20–24	25–29	30–34	35–39
Daegu	Total cases	Total	1,652	1	0	5	28	62	73	65	83
			(67.1)	(1.0)	(0.0)	(4.4)	(18.2)	(34.7)	(48.2)	(44.3)	(46.2)
		Male	939	1	0	1	16	32	30	34	55
			(77.0)	(2.0)	(0.0)	(1.7)	(19.5)	(33.0)	(36.1)	(44.6)	(61.5)
		Female	713	0	0	4	12	30	43	31	28
			(57.5)	(0.0)	(0.0)	(7.4)	(16.6)	(36.8)	(62.9)	(43.9)	(31.0)
	New cases	Total	1,333	1	0	5	28	54	58	58	68
			(54.2)	(1.0)	(0.0)	(4.4)	(18.2)	(30.3)	(38.3)	(39.5)	(37.9)
		Male	726	1	0	1	16	26	23	29	43
			(59.5)	(2.0)	(0.0)	(1.7)	(19.5)	(26.8)	(27.7)	(38.1)	(48.1)
		Female	607	0	0	4	12	28	35	29	25
			(48.9)	(0.0)	(0.0)	(7.4)	(16.6)	(34.3)	(51.2)	(41.0)	(27.7)
Incheon	Total cases	Total	1,865	1	0	4	36	82	108	105	102
			(63.9)	(0.8)	(0.0)	(3.0)	(21.0)	(39.5)	(54.4)	(52.2)	(42.6)
		Male	1,122	0	0	4	26	38	57	56	65
			(76.7)	(0.0)	(0.0)	(5.8)	(29.3)	(35.0)	(55.3)	(54.2)	(52.8)
		Female	743	1	0	0	10	44	51	49	37
			(51.1)	(1.6)	(0.0)	(0.0)	(12.1)	(44.6)	(53.4)	(50.2)	(31.7)
	New cases	Total	1,472	1	0	3	34	71	90	86	83
			(50.5)	(0.8)	(0.0)	(2.2)	(19.8)	(34.2)	(45.3)	(42.8)	(34.6)
		Male	848	0	0	3	25	34	48	45	52
			(58.0)	(0.0)	(0.0)	(4.3)	(28.1)	(31.3)	(46.6)	(43.5)	(42.3)
		Female	624	1	0	0	9	37	42	41	31
			(42.9)	(1.6)	(0.0)	(0.0)	(10.9)	(37.5)	(44.0)	(42.0)	(26.6)
Gwangju	Total cases	Total	856	1	0	0	18	35	35	47	36
			(58.7)	(1.6)	(0.0)	(0.0)	(17.1)	(31.1)	(39.2)	(50.3)	(31.4)
		Male	477	1	0	0	10	15	19	33	20
			(66.1)	(3.1)	(0.0)	(0.0)	(18.3)	(25.3)	(41.1)	(69.4)	(35.1)
		Female	379	0	0	0	8	20	16	14	16
			(51.5)	(0.0)	(0.0)	(0.0)	(15.8)	(37.6)	(37.2)	(30.5)	(27.8)
	New cases	Total	703	1	0	0	16	31	32	39	29
			(48.2)	(1.6)	(0.0)	(0.0)	(15.2)	(27.5)	(35.8)	(41.7)	(25.3)
		Male	377	1	0	0	9	13	18	28	16
			(52.2)	(3.1)	(0.0)	(0.0)	(16.5)	(21.9)	(38.9)	(58.9)	(28.1)
		Female	326	0	0	0	7	18	14	11	13
			(44.3)	(0.0)	(0.0)	(0.0)	(13.8)	(33.8)	(32.6)	(23.9)	(22.6)

(Unit : person (person/100,000 population)) – continued –

40–44	45–49	50–54	55–59	60–64	65–69	70–74	75–79	80+	unknown
84	103	129	165	116	142	142	198	256	0
(42.9)	(45.6)	(61.9)	(78.7)	(73.4)	(122.7)	(172.4)	(281.6)	(387.6)	–
56	72	84	114	74	85	80	104	101	0
(58.1)	(65.1)	(81.8)	(111.1)	(97.5)	(157.3)	(219.7)	(364.2)	(479.7)	–
28	31	45	51	42	57	62	94	155	0
(28.1)	(26.9)	(42.6)	(47.7)	(51.1)	(92.4)	(134.9)	(225.0)	(344.5)	–
66	74	109	129	85	113	122	152	211	0
(33.7)	(32.7)	(52.3)	(61.5)	(53.8)	(97.7)	(148.1)	(216.2)	(319.5)	–
42	47	69	82	51	68	67	79	82	0
(43.6)	(42.5)	(67.2)	(79.9)	(67.2)	(125.9)	(184.0)	(276.7)	(389.5)	–
24	27	40	47	34	45	55	73	129	0
(24.1)	(23.4)	(37.8)	(43.9)	(41.4)	(73.0)	(119.6)	(174.8)	(286.7)	–
125	143	177	204	158	122	102	146	250	0
(52.3)	(54.3)	(71.2)	(82.1)	(92.9)	(110.8)	(121.3)	(219.2)	(360.7)	–
84	95	120	144	117	74	65	68	109	0
(68.7)	(72.2)	(96.3)	(114.5)	(138.1)	(137.1)	(167.8)	(249.0)	(513.1)	–
41	48	57	60	41	48	37	78	141	0
(35.1)	(36.4)	(46.0)	(48.9)	(48.0)	(85.5)	(81.5)	(198.6)	(293.3)	–
102	115	136	153	118	83	79	116	202	0
(42.6)	(43.7)	(54.7)	(61.6)	(69.4)	(75.4)	(93.9)	(174.2)	(291.4)	–
67	72	90	103	84	48	47	49	81	0
(54.8)	(54.7)	(72.2)	(81.9)	(99.1)	(88.9)	(121.3)	(179.4)	(381.3)	–
35	43	46	50	34	35	32	67	121	0
(29.9)	(32.6)	(37.1)	(40.7)	(39.8)	(62.3)	(70.5)	(170.6)	(251.7)	–
47	59	66	83	55	68	58	90	158	0
(38.7)	(45.1)	(57.9)	(76.8)	(72.4)	(116.0)	(129.9)	(247.9)	(445.3)	–
23	39	42	56	39	41	36	43	60	0
(38.1)	(60.2)	(73.8)	(106.3)	(106.7)	(149.4)	(177.1)	(286.0)	(547.7)	–
24	20	24	27	16	27	22	47	98	0
(39.2)	(30.3)	(42.0)	(48.7)	(40.6)	(86.7)	(90.4)	(221.0)	(399.5)	–
41	49	52	70	34	52	48	76	133	0
(33.7)	(37.5)	(45.6)	(64.7)	(44.8)	(88.7)	(107.5)	(209.4)	(374.8)	–
18	33	30	46	24	30	28	34	49	0
(29.8)	(51.0)	(52.7)	(87.3)	(65.6)	(109.3)	(137.8)	(226.1)	(447.3)	–
23	16	22	24	10	22	20	42	84	0
(37.6)	(24.2)	(38.5)	(43.3)	(25.4)	(70.6)	(82.2)	(197.5)	(342.5)	–

[Table 3] (New) TB notification cases and rates by province, gender and age, 2017

Province		Age / Gender	Total	0–4	5–9	10–14	15–19	20–24	25–29	30–34	35–39
Daejeon	Total cases	Total	846	0	0	2	16	43	49	37	33
			(56.5)	(0.0)	(0.0)	(2.7)	(16.5)	(38.4)	(48.8)	(36.7)	(28.3)
		Male	521	0	0	0	7	25	25	23	18
			(69.7)	(0.0)	(0.0)	(0.0)	(13.9)	(42.4)	(46.7)	(44.3)	(30.8)
		Female	325	0	0	2	9	18	24	14	15
			(43.4)	(0.0)	(0.0)	(5.5)	(19.4)	(33.9)	(51.2)	(28.7)	(25.7)
	New cases	Total	681	0	0	2	14	36	41	32	26
			(45.5)	(0.0)	(0.0)	(2.7)	(14.5)	(32.1)	(40.8)	(31.8)	(22.3)
		Male	404	0	0	0	6	22	23	21	13
			(54.0)	(0.0)	(0.0)	(0.0)	(11.9)	(37.3)	(42.9)	(40.4)	(22.2)
		Female	277	0	0	2	8	14	18	11	13
			(37.0)	(0.0)	(0.0)	(5.5)	(17.3)	(26.4)	(38.4)	(22.6)	(22.3)
Ulsan	Total cases	Total	695	0	0	4	13	24	23	24	36
			(59.8)	(0.0)	(0.0)	(7.2)	(17.5)	(28.7)	(31.1)	(30.8)	(37.9)
		Male	397	0	0	1	11	12	11	16	22
			(66.4)	(0.0)	(0.0)	(3.4)	(27.9)	(25.5)	(26.6)	(38.9)	(44.4)
		Female	298	0	0	3	2	12	12	8	14
			(52.8)	(0.0)	(0.0)	(11.4)	(5.8)	(32.8)	(36.9)	(21.7)	(30.8)
	New cases	Total	556	0	0	4	11	22	17	20	32
			(47.8)	(0.0)	(0.0)	(7.2)	(14.8)	(26.3)	(23.0)	(25.6)	(33.6)
		Male	302	0	0	1	9	11	9	13	18
			(50.5)	(0.0)	(0.0)	(3.4)	(22.8)	(23.4)	(21.8)	(31.6)	(36.3)
		Female	254	0	0	3	2	11	8	7	14
			(45.0)	(0.0)	(0.0)	(11.4)	(5.8)	(30.1)	(24.6)	(19.0)	(30.8)
Sejong	Total cases	Total	137	0	0	0	2	7	6	6	7
			(52.6)	(0.0)	(0.0)	(0.0)	(14.9)	(51.9)	(40.3)	(28.6)	(25.0)
		Male	92	0	0	0	0	6	3	4	7
			(70.7)	(0.0)	(0.0)	(0.0)	(0.0)	(84.4)	(41.5)	(39.6)	(51.0)
		Female	45	0	0	0	2	1	3	2	0
			(34.5)	(0.0)	(0.0)	(0.0)	(30.5)	(15.7)	(39.3)	(18.4)	(0.0)
	New cases	Total	92	0	0	0	2	5	4	6	5
			(35.3)	(0.0)	(0.0)	(0.0)	(14.9)	(37.1)	(26.9)	(28.6)	(17.8)
		Male	55	0	0	0	0	4	1	4	5
			(42.3)	(0.0)	(0.0)	(0.0)	(0.0)	(56.3)	(13.8)	(39.6)	(36.5)
		Female	37	0	0	0	2	1	3	2	0
			(28.4)	(0.0)	(0.0)	(0.0)	(30.5)	(15.7)	(39.3)	(18.4)	(0.0)

(Unit : person (person/100,000 population)) – continued –

40–44	45–49	50–54	55–59	60–64	65–69	70–74	75–79	80+	unknown
49	63	64	87	87	58	69	67	122	0
(40.0)	(46.3)	(54.8)	(72.8)	(99.4)	(98.4)	(158.9)	(188.3)	(333.9)	–
33	39	44	62	58	47	41	46	53	0
(54.3)	(57.7)	(75.2)	(105.2)	(134.9)	(164.6)	(205.2)	(307.7)	(452.5)	–
16	24	20	25	29	11	28	21	69	0
(25.9)	(35.0)	(34.3)	(41.3)	(65.2)	(36.2)	(119.4)	(101.8)	(277.9)	–
37	48	46	69	71	48	54	55	102	0
(30.2)	(35.3)	(39.4)	(57.7)	(81.1)	(81.4)	(124.4)	(154.6)	(279.1)	–
23	30	28	49	47	39	30	35	38	0
(37.9)	(44.4)	(47.9)	(83.1)	(109.3)	(136.6)	(150.1)	(234.1)	(324.4)	–
14	18	18	20	24	9	24	20	64	0
(22.6)	(26.3)	(30.9)	(33.0)	(53.9)	(29.6)	(102.4)	(96.9)	(257.7)	–
46	56	69	68	62	57	59	56	98	0
(49.2)	(51.3)	(66.3)	(67.6)	(90.0)	(131.9)	(213.1)	(268.0)	(492.0)	–
21	37	45	49	37	33	39	22	41	0
(44.5)	(68.4)	(83.3)	(94.8)	(106.0)	(149.9)	(295.3)	(253.9)	(747.6)	–
25	19	24	19	25	24	20	34	57	0
(54.0)	(34.5)	(47.9)	(38.9)	(73.6)	(113.2)	(138.1)	(278.0)	(394.9)	–
33	45	53	58	48	45	40	47	81	0
(35.3)	(41.2)	(50.9)	(57.7)	(69.7)	(104.1)	(144.5)	(224.9)	(406.6)	–
16	30	32	41	27	22	24	16	33	0
(33.9)	(55.5)	(59.3)	(79.4)	(77.4)	(100.0)	(181.7)	(184.6)	(601.8)	–
17	15	21	17	21	23	16	31	48	0
(36.7)	(27.2)	(41.9)	(34.8)	(61.8)	(108.5)	(110.5)	(253.5)	(332.5)	–
5	9	9	11	19	8	9	15	24	0
(19.1)	(41.4)	(56.8)	(71.2)	(164.6)	(100.8)	(153.8)	(282.2)	(388.0)	–
4	7	9	9	15	6	1	11	10	0
(29.6)	(59.1)	(108.0)	(114.1)	(261.6)	(156.5)	(37.7)	(505.2)	(527.8)	–
1	2	0	2	4	2	8	4	14	0
(7.9)	(20.2)	(0.0)	(26.4)	(68.9)	(48.8)	(250.0)	(127.5)	(326.3)	–
2	7	6	4	12	6	7	8	18	0
(7.6)	(32.2)	(37.9)	(25.9)	(104.0)	(75.6)	(119.6)	(150.5)	(291.0)	–
2	5	6	3	10	5	0	4	6	0
(14.8)	(42.2)	(72.0)	(38.0)	(174.4)	(130.4)	(0.0)	(183.7)	(316.7)	–
0	2	0	1	2	1	7	4	12	0
(0.0)	(20.2)	(0.0)	(13.2)	(34.4)	(24.4)	(218.8)	(127.5)	(279.7)	–

[Table 3] (New) TB notification cases and rates by province, gender and age, 2017

Province		Age / Gender	Total	0–4	5–9	10–14	15–19	20–24	25–29	30–34	35–39
Gyeonggi	Total cases	Total	7,818	3	4	20	163	383	482	410	457
			(61.6)	(0.5)	(0.6)	(3.1)	(20.8)	(43.8)	(58.7)	(47.3)	(42.9)
		Male	4,770	2	3	12	89	223	255	240	255
			(74.8)	(0.7)	(0.9)	(3.7)	(21.9)	(48.7)	(59.5)	(54.1)	(47.3)
		Female	3,048	1	1	8	74	160	227	170	202
			(48.3)	(0.4)	(0.3)	(2.6)	(19.6)	(38.5)	(57.8)	(40.2)	(38.5)
	New cases	Total	6,130	3	4	20	151	328	415	326	371
			(48.3)	(0.5)	(0.6)	(3.1)	(19.3)	(37.5)	(50.5)	(37.6)	(34.9)
		Male	3,582	2	3	12	84	189	215	187	198
			(56.2)	(0.7)	(0.9)	(3.7)	(20.7)	(41.2)	(50.1)	(42.2)	(36.7)
		Female	2,548	1	1	8	67	139	200	139	173
			(40.4)	(0.4)	(0.3)	(2.6)	(17.7)	(33.4)	(50.9)	(32.8)	(32.9)
Gangwon	Total cases	Total	1,502	0	1	5	47	23	45	39	60
			(97.7)	(0.0)	(1.5)	(7.3)	(51.3)	(22.4)	(54.8)	(48.0)	(60.1)
		Male	859	0	1	3	38	10	21	23	36
			(111.0)	(0.0)	(3.0)	(8.5)	(78.9)	(17.3)	(45.4)	(53.3)	(70.8)
		Female	643	0	0	2	9	13	24	16	24
			(84.3)	(0.0)	(0.0)	(6.1)	(20.7)	(29.0)	(67.1)	(41.9)	(49.0)
	New cases	Total	1,165	0	1	5	45	18	40	32	42
			(75.8)	(0.0)	(1.5)	(7.3)	(49.1)	(17.5)	(48.7)	(39.3)	(42.1)
		Male	652	0	1	3	38	7	18	18	27
			(84.2)	(0.0)	(3.0)	(8.5)	(78.9)	(12.1)	(38.9)	(41.7)	(53.1)
		Female	513	0	0	2	7	11	22	14	15
			(67.3)	(0.0)	(0.0)	(6.1)	(16.1)	(24.5)	(61.5)	(36.7)	(30.6)
Chungbuk	Total cases	Total	1,154	2	1	2	12	38	39	34	47
			(72.9)	(3.0)	(1.4)	(2.7)	(12.5)	(35.5)	(42.7)	(35.3)	(41.9)
		Male	669	0	1	0	6	22	22	17	23
			(83.8)	(0.0)	(2.6)	(0.0)	(12.0)	(37.7)	(43.9)	(33.4)	(39.5)
		Female	485	2	0	2	6	16	17	17	24
			(61.9)	(6.2)	(0.0)	(5.7)	(13.1)	(33.0)	(41.3)	(37.3)	(44.4)
	New cases	Total	844	2	1	2	12	32	33	24	38
			(53.3)	(3.0)	(1.4)	(2.7)	(12.5)	(29.9)	(36.2)	(24.9)	(33.8)
		Male	464	0	1	0	6	19	19	10	19
			(58.1)	(0.0)	(2.6)	(0.0)	(12.0)	(32.5)	(37.9)	(19.7)	(32.6)
		Female	380	2	0	2	6	13	14	14	19
			(48.5)	(6.2)	(0.0)	(5.7)	(13.1)	(26.8)	(34.0)	(30.7)	(35.2)

(Unit : person (person/100,000 population)) − continued −

40–44	45–49	50–54	55–59	60–64	65–69	70–74	75–79	80+	unknown
565	636	664	799	641	488	505	629	969	0
(51.3)	(53.6)	(64.3)	(80.8)	(93.6)	(108.3)	(140.8)	(214.3)	(324.6)	−
336	409	477	568	454	331	317	361	438	0
(60.3)	(68.3)	(90.1)	(112.5)	(132.8)	(152.3)	(193.3)	(291.2)	(447.6)	−
229	227	187	231	187	157	188	268	531	0
(42.0)	(38.7)	(37.1)	(47.7)	(54.5)	(67.4)	(96.5)	(158.1)	(264.6)	−
442	487	496	593	479	375	379	499	762	0
(40.1)	(41.1)	(48.0)	(60.0)	(69.9)	(83.2)	(105.7)	(170.0)	(255.3)	−
259	292	341	406	324	244	223	269	334	0
(46.5)	(48.8)	(64.4)	(80.4)	(94.8)	(112.3)	(136.0)	(217.0)	(341.3)	−
183	195	155	187	155	131	156	230	428	0
(33.6)	(33.2)	(30.8)	(38.6)	(45.2)	(56.2)	(80.1)	(135.7)	(213.3)	−
50	88	97	134	131	123	178	188	293	0
(44.5)	(68.1)	(77.8)	(93.6)	(116.0)	(168.2)	(256.3)	(295.5)	(461.9)	−
36	64	73	101	83	67	95	102	106	0
(62.2)	(94.8)	(113.1)	(140.6)	(146.2)	(191.9)	(301.1)	(384.6)	(529.4)	−
14	24	24	33	48	56	83	86	187	0
(25.7)	(38.9)	(39.9)	(46.3)	(85.4)	(146.5)	(219.1)	(231.8)	(430.7)	−
43	69	67	97	102	86	137	145	236	0
(38.3)	(53.4)	(53.7)	(67.8)	(90.3)	(117.6)	(197.3)	(227.9)	(372.0)	−
30	49	51	73	64	45	67	75	86	0
(51.9)	(72.6)	(79.0)	(101.6)	(112.7)	(128.9)	(212.4)	(282.8)	(429.5)	−
13	20	16	24	38	41	70	70	150	0
(23.9)	(32.4)	(26.6)	(33.7)	(67.6)	(107.3)	(184.8)	(188.7)	(345.5)	−
60	60	79	109	84	66	112	150	259	0
(50.1)	(44.3)	(62.5)	(79.7)	(80.2)	(94.1)	(189.6)	(270.4)	(432.6)	−
39	38	54	81	58	41	71	80	116	0
(63.1)	(54.6)	(82.2)	(116.2)	(110.4)	(119.5)	(266.3)	(353.2)	(623.6)	−
21	22	25	28	26	25	41	70	143	0
(36.2)	(33.5)	(41.1)	(41.7)	(49.8)	(69.9)	(126.5)	(213.2)	(346.5)	−
42	34	52	72	58	53	89	119	181	0
(35.1)	(25.1)	(41.1)	(52.6)	(55.4)	(75.6)	(150.7)	(214.5)	(302.3)	−
29	20	36	51	38	32	50	62	72	0
(46.9)	(28.7)	(54.8)	(73.2)	(72.3)	(93.2)	(187.6)	(273.8)	(387.1)	−
13	14	16	21	20	21	39	57	109	0
(22.4)	(21.3)	(26.3)	(31.3)	(38.3)	(58.7)	(120.3)	(173.6)	(264.1)	−

[Table 3] (New) TB notification cases and rates by province, gender and age, 2017

Province		Age / Gender	Total	0-4	5-9	10-14	15-19	20-24	25-29	30-34	35-39
Chungnam	Total cases	Total	1,720	1	0	2	23	55	52	46	52
			(82.2)	(1.1)	(0.0)	(2.0)	(18.9)	(42.7)	(44.2)	(34.6)	(32.8)
		Male	1,062	1	0	2	9	28	32	27	30
			(99.9)	(2.1)	(0.0)	(3.9)	(14.1)	(40.4)	(49.9)	(37.9)	(35.5)
		Female	658	0	0	0	14	27	20	19	22
			(63.9)	(0.0)	(0.0)	(0.0)	(24.1)	(45.4)	(37.3)	(30.8)	(29.8)
	New cases	Total	1,359	1	0	2	22	46	46	35	38
			(65.0)	(1.1)	(0.0)	(2.0)	(18.1)	(35.7)	(39.1)	(26.3)	(24.0)
		Male	801	1	0	2	9	23	29	17	18
			(75.3)	(2.1)	(0.0)	(3.9)	(14.1)	(33.2)	(45.2)	(23.9)	(21.3)
		Female	558	0	0	0	13	23	17	18	20
			(54.2)	(0.0)	(0.0)	(0.0)	(22.3)	(38.7)	(31.7)	(29.2)	(27.1)
Jeonbuk	Total cases	Total	1,352	0	0	1	17	36	30	38	49
			(73.2)	(0.0)	(0.0)	(1.1)	(14.6)	(29.1)	(31.6)	(38.8)	(40.4)
		Male	786	0	0	0	10	17	13	15	26
			(85.5)	(0.0)	(0.0)	(0.0)	(16.5)	(25.6)	(25.6)	(29.6)	(41.6)
		Female	566	0	0	1	7	19	17	23	23
			(61.0)	(0.0)	(0.0)	(2.4)	(12.5)	(33.1)	(38.4)	(48.7)	(39.2)
	New cases	Total	1,025	0	0	1	17	26	26	29	40
			(55.5)	(0.0)	(0.0)	(1.1)	(14.6)	(21.0)	(27.4)	(29.6)	(33.0)
		Male	562	0	0	0	10	14	12	9	20
			(61.2)	(0.0)	(0.0)	(0.0)	(16.5)	(21.1)	(23.6)	(17.7)	(32.0)
		Female	463	0	0	1	7	12	14	20	20
			(49.9)	(0.0)	(0.0)	(2.4)	(12.5)	(20.9)	(31.6)	(42.3)	(34.1)
Jeonnam	Total cases	Total	1,914	1	1	3	27	31	30	48	55
			(101.4)	(1.4)	(1.2)	(3.6)	(23.8)	(26.4)	(33.9)	(50.0)	(46.6)
		Male	1,069	1	0	1	18	15	13	23	33
			(113.2)	(2.7)	(0.0)	(2.3)	(30.4)	(23.4)	(27.3)	(45.7)	(52.9)
		Female	845	0	1	2	9	16	17	25	22
			(89.6)	(0.0)	(2.5)	(5.0)	(16.7)	(29.9)	(41.6)	(54.8)	(39.4)
	New cases	Total	1,503	1	1	3	26	30	25	40	43
			(79.6)	(1.4)	(1.2)	(3.6)	(23.0)	(25.5)	(28.2)	(41.7)	(36.4)
		Male	788	1	0	1	17	14	9	19	24
			(83.5)	(2.7)	(0.0)	(2.3)	(28.7)	(21.9)	(18.9)	(37.7)	(38.5)
		Female	715	0	1	2	9	16	16	21	19
			(75.8)	(0.0)	(2.5)	(5.0)	(16.7)	(29.9)	(39.1)	(46.0)	(34.1)

(Unit : person (person/100,000 population)) – continued –

40–44	45–49	50–54	55–59	60–64	65–69	70–74	75–79	80+	unknown
88	91	124	133	140	114	141	222	436	0
(55.0)	(52.7)	(79.5)	(79.4)	(108.6)	(113.5)	(175.7)	(280.7)	(466.0)	–
55	67	85	94	105	77	102	131	217	0
(64.9)	(72.7)	(102.7)	(109.2)	(162.9)	(156.9)	(278.9)	(409.4)	(703.6)	–
33	24	39	39	35	37	39	91	219	0
(43.8)	(29.8)	(53.2)	(47.8)	(54.3)	(72.0)	(89.3)	(193.3)	(349.2)	–
71	71	97	98	99	84	102	180	367	0
(44.4)	(41.1)	(62.2)	(58.5)	(76.8)	(83.6)	(127.1)	(227.6)	(392.3)	–
42	50	63	71	73	54	67	103	179	0
(49.6)	(54.2)	(76.2)	(82.5)	(113.3)	(110.0)	(183.2)	(321.9)	(580.4)	–
29	21	34	27	26	30	35	77	188	0
(38.5)	(26.1)	(46.4)	(33.1)	(40.3)	(58.4)	(80.1)	(163.5)	(299.7)	–
64	71	88	118	107	94	140	174	325	0
(46.8)	(46.1)	(61.4)	(76.9)	(88.5)	(94.8)	(173.1)	(226.7)	(374.7)	–
45	46	60	90	80	57	88	95	144	0
(63.7)	(57.1)	(80.4)	(116.7)	(133.2)	(119.7)	(244.5)	(313.3)	(537.9)	–
19	25	28	28	27	37	52	79	181	0
(28.7)	(34.1)	(40.7)	(36.7)	(44.4)	(71.8)	(115.9)	(170.2)	(301.9)	–
42	60	57	88	75	71	107	128	258	0
(30.7)	(39.0)	(39.8)	(57.4)	(62.1)	(71.6)	(132.3)	(166.8)	(297.5)	–
27	36	37	65	58	41	64	67	102	0
(38.2)	(44.7)	(49.6)	(84.3)	(96.6)	(86.1)	(177.8)	(221.0)	(381.0)	–
15	24	20	23	17	30	43	61	156	0
(22.7)	(32.7)	(29.1)	(30.2)	(28.0)	(58.2)	(95.8)	(131.4)	(260.2)	–
72	104	124	139	116	137	194	340	492	0
(55.8)	(68.5)	(82.2)	(86.1)	(93.9)	(126.1)	(206.5)	(352.0)	(484.2)	–
48	77	90	101	80	93	114	174	188	0
(69.4)	(93.3)	(110.3)	(121.3)	(128.5)	(175.6)	(277.7)	(470.0)	(645.2)	–
24	27	34	38	36	44	80	166	304	0
(40.1)	(38.9)	(49.1)	(48.7)	(58.7)	(79.0)	(151.3)	(278.7)	(419.4)	–
62	74	89	102	89	122	159	249	388	0
(48.1)	(48.7)	(59.0)	(63.2)	(72.0)	(112.3)	(169.3)	(257.8)	(381.8)	–
41	54	60	70	56	80	85	119	138	0
(59.3)	(65.5)	(73.5)	(84.1)	(90.0)	(151.1)	(207.1)	(321.5)	(473.6)	–
21	20	29	32	33	42	74	130	250	0
(35.1)	(28.8)	(41.9)	(41.0)	(53.8)	(75.4)	(139.9)	(218.2)	(344.9)	–

[Table 3] (New) TB notification cases and rates by province, gender and age, 2017

Province		Age / Gender	Total	0–4	5–9	10–14	15–19	20–24	25–29	30–34	35–39
Chungbuk	Total cases	Total	2,527	1	0	7	16	55	62	63	77
			(94.4)	(0.9)	(0.0)	(6.3)	(10.7)	(33.3)	(44.1)	(41.8)	(42.5)
		Male	1,432	0	0	6	15	34	33	38	46
			(106.4)	(0.0)	(0.0)	(10.4)	(18.9)	(37.1)	(41.9)	(47.6)	(48.5)
		Female	1,095	1	0	1	1	21	29	25	31
			(82.2)	(1.9)	(0.0)	(1.9)	(1.4)	(28.6)	(46.9)	(35.3)	(35.9)
	New cases	Total	1,978	1	0	7	15	44	57	49	60
			(73.9)	(0.9)	(0.0)	(6.3)	(10.1)	(26.6)	(40.5)	(32.5)	(33.1)
		Male	1,062	0	0	6	14	25	30	29	34
			(78.9)	(0.0)	(0.0)	(10.4)	(17.7)	(27.3)	(38.1)	(36.3)	(35.9)
		Female	916	1	0	1	1	19	27	20	26
			(68.8)	(1.9)	(0.0)	(1.9)	(1.4)	(25.9)	(43.6)	(28.3)	(30.1)
Gyeongnam	Total cases	Total	2,395	0	1	6	38	48	75	96	103
			(71.4)	(0.0)	(0.6)	(3.8)	(18.6)	(22.2)	(41.8)	(47.7)	(40.3)
		Male	1,445	0	0	2	25	19	45	53	66
			(85.5)	(0.0)	(0.0)	(2.4)	(23.2)	(15.8)	(46.0)	(50.3)	(49.8)
		Female	950	0	1	4	13	29	30	43	37
			(57.0)	(0.0)	(1.3)	(5.3)	(13.5)	(30.1)	(36.8)	(44.8)	(30.0)
	New cases	Total	1,853	0	1	6	36	45	63	77	80
			(55.2)	(0.0)	(0.6)	(3.8)	(17.6)	(20.8)	(35.1)	(38.3)	(31.3)
		Male	1,067	0	0	2	23	18	36	41	50
			(63.2)	(0.0)	(0.0)	(2.4)	(21.3)	(15.0)	(36.8)	(38.9)	(37.7)
		Female	786	0	1	4	13	27	27	36	30
			(47.1)	(0.0)	(1.3)	(5.3)	(13.5)	(28.0)	(33.1)	(37.5)	(24.3)
Jeju	Total cases	Total	486	0	0	2	9	22	21	24	31
			(75.5)	(0.0)	(0.0)	(5.8)	(21.9)	(50.8)	(60.7)	(63.4)	(63.6)
		Male	304	0	0	0	8	13	9	11	14
			(93.8)	(0.0)	(0.0)	(0.0)	(37.3)	(56.6)	(49.8)	(57.1)	(56.4)
		Female	182	0	0	2	1	9	12	13	17
			(56.9)	(0.0)	(0.0)	(12.4)	(5.1)	(44.2)	(72.7)	(70.0)	(71.0)
	New cases	Total	380	0	0	1	9	20	17	22	23
			(59.0)	(0.0)	(0.0)	(2.9)	(21.9)	(46.2)	(49.2)	(58.1)	(47.2)
		Male	228	0	0	0	8	11	7	10	11
			(70.3)	(0.0)	(0.0)	(0.0)	(37.3)	(47.9)	(38.7)	(51.9)	(44.3)
		Female	152	0	0	1	1	9	10	12	12
			(47.5)	(0.0)	(0.0)	(6.2)	(5.1)	(44.2)	(60.6)	(64.6)	(50.1)

(Unit : person (person/100,000 population))

40–44	45–49	50–54	55–59	60–64	65–69	70–74	75–79	80+	unknown
87	117	139	206	186	206	261	381	663	0
(45.3)	(52.7)	(63.9)	(86.3)	(96.2)	(139.0)	(229.8)	(336.6)	(534.8)	–
65	85	101	155	118	139	140	172	285	0
(65.2)	(73.2)	(89.3)	(128.1)	(122.9)	(192.4)	(278.4)	(387.3)	(758.8)	–
22	32	38	51	68	67	121	209	378	0
(23.8)	(30.2)	(36.4)	(43.3)	(69.8)	(88.3)	(191.3)	(303.9)	(437.4)	–
65	92	91	155	144	161	207	312	518	0
(33.8)	(41.4)	(41.9)	(64.9)	(74.4)	(108.7)	(182.3)	(275.7)	(417.8)	–
46	65	64	115	84	102	104	132	212	0
(46.1)	(56.0)	(56.6)	(95.1)	(87.5)	(141.2)	(206.8)	(297.2)	(564.4)	–
19	27	27	40	60	59	103	180	306	0
(20.6)	(25.5)	(25.9)	(34.0)	(61.6)	(77.7)	(162.8)	(261.7)	(354.1)	–
116	216	197	234	210	174	181	243	457	0
(43.7)	(71.9)	(72.3)	(81.4)	(97.5)	(112.9)	(157.4)	(230.2)	(401.4)	–
70	142	149	185	159	126	108	120	176	0
(51.3)	(92.2)	(105.8)	(127.0)	(148.1)	(168.4)	(212.5)	(298.3)	(548.3)	–
46	74	48	49	51	48	73	123	281	0
(35.7)	(50.6)	(36.4)	(34.5)	(47.2)	(60.5)	(113.8)	(188.2)	(343.8)	–
94	144	147	179	156	132	139	186	368	0
(35.4)	(48.0)	(53.9)	(62.2)	(72.4)	(85.6)	(120.9)	(176.2)	(323.3)	–
57	90	110	137	112	94	79	85	133	0
(41.8)	(58.4)	(78.1)	(94.1)	(104.3)	(125.6)	(155.5)	(211.3)	(414.3)	–
37	54	37	42	44	38	60	101	235	0
(28.7)	(36.9)	(28.1)	(29.6)	(40.8)	(47.9)	(93.5)	(154.6)	(287.5)	–
40	44	52	52	46	32	23	31	57	0
(75.2)	(74.4)	(99.5)	(105.7)	(126.2)	(120.9)	(103.5)	(164.5)	(254.3)	–
25	31	41	39	31	25	15	16	26	0
(90.1)	(100.1)	(148.7)	(154.6)	(170.7)	(195.3)	(145.7)	(207.4)	(428.4)	–
15	13	11	13	15	7	8	15	31	0
(58.9)	(46.2)	(44.6)	(54.3)	(82.0)	(51.2)	(67.0)	(134.7)	(189.6)	–
32	33	38	39	27	27	15	25	52	0
(60.2)	(55.8)	(72.7)	(79.3)	(74.1)	(102.0)	(67.5)	(132.6)	(232.0)	–
22	22	30	28	16	20	9	12	22	0
(79.3)	(71.0)	(108.8)	(111.0)	(88.1)	(156.3)	(87.4)	(155.5)	(362.5)	–
10	11	8	11	11	7	6	13	30	0
(39.3)	(39.1)	(32.4)	(45.9)	(60.1)	(51.2)	(50.3)	(116.8)	(183.5)	–

[Table 4] (New) TB notification cases by regions, 2017

(Unit : person) – continued –

Province	District	Total cases	New cases
Whole Country		36,044	28,161
Seoul		6,663	5,226
	Gangnamgu	227	185
	Gangdonggu	242	192
	Gangbukgu	257	194
	Gangseogu	360	267
	Gwanakgu	457	364
	Gwangjingu	248	202
	Gurogu	340	262
	Geumcheongu	234	168
	Nowongu	326	259
	Dobonggu	210	173
	Dongdaemungu	289	222
	Dongjakgu	285	226
	Mapogu	215	171
	Seodaemungu	189	156
	Seochogu	169	143
	Seongdonggu	202	161
	Seongbukgu	298	238
	Songpagu	330	265
	Yangcheongu	255	208
	Yeongdeungpogu	365	283
	Yongsangu	187	140
	Eunpyeonggu	342	280
	Jongnogu	151	102
	Junggu	116	80
	Jungnanggu	369	285
Busan		2,462	1,861
	Gangseogu	64	51
	Gumjunggu	169	127
	Gijanggun	101	73
	Namgu	175	135
	Donggu	107	77
	Dongnaegu	163	125
	Busanjingu	289	220
Busan	Bukgu	198	148
	Sasanggu	192	155
	Sahagu	245	180
	Seogu	113	76
	Suyeonggu	113	88
	Yeonjegu	143	111
	Yeongdogu	121	94
	Junggu	44	37
	Haeundaegu	225	164
Daegu		1,652	1,333
	Namgu	135	102
	Dalseogu	377	309
	Dalseonggun	122	102
	Donggu	273	217
	Bukgu	260	219
	Seogu	209	164
	Suseonggu	229	181
	Junggu	47	39
Incheon		1,865	1,472
	Ganghwagun	51	37
	Gyeyanggu	194	145
	Namgu	317	249
	Namdonggu	308	253
	Donggu	60	50
	Bupyeonggu	378	293
	Seogu	297	242
	Yeonsugu	164	131
	Ongjingun	21	16
	Junggu	75	56
Gwangju		856	703
	Gwangsangu	216	181
	Namgu	127	99
	Donggu	77	63
	Bukgu	279	226
	Seogu	157	134

[Table 4] (New) TB notification cases by regions, 2017

(Unit : person) – continued –

Province	District	Total cases	New cases	Province	District	Total cases	New cases
Daejeon		846	681	Gyeonggi	Ansansi Sangnokgu	277	219
	Daedeokgu	102	83		Anseongsi	166	126
	Donggu	159	124		Anyangsi Dongangu	151	121
	Seogu	240	193		Anyangsi Manangu	171	137
	Yuseonggu	147	125		Yangjusi	134	107
	Junggu	198	156		Yangpyeonggun	67	54
Ulsan		695	556		Yeojusi	101	75
	Namgu	199	158		Yeoncheongun	35	28
	Donggu	84	69		Osansi	125	102
	Bukgu	97	74		Yonginsi Giheunggu	160	138
	Uljugun	148	128		Yonginsi Sujigu	115	100
	Junggu	167	127		Yonginsi Cheoingu	198	149
Sejong		137	92		Uiwangsi	72	59
Gyeonggi		7,818	6,130		Uijeongbusi	310	237
	Gapyeonggun	57	38		Icheonsi	128	92
	Goyangsi Deogyanggu	238	195		Pajusi	214	160
	Goyangsi Ilsandonggu	151	125		Pyeongtaeksi	283	215
	Goyangsi Ilsanseogu	151	127		Pocheonsi	135	92
	Gwacheonsi	35	30		Hanamsi	108	92
	Gwangmyeongsi	215	161		Hwaseongsi	339	272
	Gwangjusi	208	169	Gangwon		1,502	1,165
	Gurisi	129	100		Gangneungsi	228	183
	Gunposi	152	125		Goseonggun	29	18
	Gimposi	231	190		Donghaesi	98	72
	Namyangjusi	409	308		Samcheoksi	124	89
	Dongducheonsi	75	58		Sokchosi	58	41
	Bucheonsi	576	453		Yanggugun	10	6
	Seongnamsi Bundanggu	200	164		Yangyanggun	30	23
	Seongnamsi Sujeonggu	204	169		Yeongwolgun	72	56
	Seongnamsi Jungwongu	193	155		Wonjusi	219	176
	Seongnamsi Gwonseongu	198	155		Injegun	32	27
	Suwonsi Yeongtonggu	132	104		Jeongseongun	79	57
	Suwonsi Jangangu	140	116		Cheorwongun	37	30
	Suwonsi Paldalgu	160	114		Chuncheonsi	199	163
	Siheungsi	333	235		Taebaeksi	77	54
	Ansansi Danwongu	342	264		Pyeongchanggun	53	38

[Table 4] (New) TB notification cases by regions, 2017

(Unit : person) – continued –

Province	District	Total cases	New cases
Gangwon	Hongcheongun	87	71
	Hwacheongun	24	23
	Hoengseonggun	46	38
Chungbuk		1,154	844
	Goesangun	43	29
	Danyanggun	32	21
	Boeungun	41	32
	Yeongdonggun	63	50
	Okcheongun	60	52
	Eumseonggun	109	77
	Jecheonsi	125	88
	Jeungpyeonggun	34	27
	Jincheongun	70	52
	Cheongjusi Sangdanggu	95	65
	Cheongjusi Seowongu	104	76
	Cheongjusi Cheongwongu	105	86
	Cheongjusi Heungdeokgu	130	92
	Chungjusi	143	97
Chungnam		1,720	1,359
	Gyeryongsi	14	11
	Gongjusi	113	88
	Geumsangun	66	53
	Nonsansi	134	111
	Dangjinsi	130	100
	Boryeongsi	124	89
	Buyeogun	92	73
	Seosansi	135	103
	Seocheongun	57	46
	Asansi	207	162
	Yesangun	92	80
	Cheonansi Dongnamgu	143	110
	Cheonansi Seobukgu	195	156
	Cheongyanggun	37	33
	Taeangun	95	75
	Hongseonggun	86	69
Jeonbuk		1,352	1,025
Jeonbuk	Gochanggun	69	49
	Gunsansi	148	108
	Gimjesi	102	78
	Namwonsi	63	48
	Mujugun	29	24
	Buangun	84	61
	Sunchanggun	42	35
	Wanjugun	83	61
	Iksansi	244	175
	Imsilgun	21	17
	Jangsugun	17	12
	Jeonjusi	306	251
	Jeongeupsi	126	92
	Jinangun	18	14
Jeonnam		1,914	1,503
	Gangjingun	71	56
	Goheunggun	124	105
	Gokseonggun	32	21
	Gwangyangsi	77	66
	Guryegun	29	24
	Najusi	118	93
	Damyanggun	39	29
	Mokposi	202	145
	Muangun	107	86
	Boseonggun	53	42
	Suncheonsi	156	125
	Sinangun	88	74
	Yeosusi	162	127
	Yeonggwanggun	66	50
	Yeongamgun	89	72
	Wandogun	75	60
	Jangseonggun	53	45
	Jangheunggun	50	34
	Jindogun	54	41
	Hampyeonggun	81	71
	Haenamgun	115	79

[Table 4] (New) TB notification cases by regions, 2017

(Unit : person)

Province	District	Total cases	New cases
Jeonnam	Hwasungun	73	58
Gyeongbuk		2,527	1,978
	Gyeongsansi	186	154
	Gyeongjusi	278	215
	Goryeonggun	54	40
	Gumisi	223	177
	Gunwigun	36	30
	Gimcheonsi	118	96
	Mungyeongsi	72	63
	Bonghwagun	44	33
	Sangjusi	91	69
	Seongjugun	45	37
	Andongsi	147	106
	Yeongdeokgun	66	51
	Yeongyanggun	33	26
	Yeongjusi	93	66
	Yeongcheonsi	165	129
	Yecheongun	51	40
	Ulleunggun	9	6
	Uljingun	81	68
	Uiseonggun	115	89
	Cheongdogun	39	29
	Cheongsonggun	64	46
	Chilgokgun	85	70
	Pohangsi Namgu	188	145
	Pohangsi Bukgu	244	193
Gyeongnam		2,395	1,853
	Geojesi	124	99
	Geochanggun	60	47
	Goseonggun	80	55
	Gimhaesi	328	242
	Namhaegun	46	39
	Miryangsi	124	88
	Sacheonsi	100	79
	Sancheonggun	33	27
	Yangsansi	179	141
Gyeongnam	Uiryeonggun	47	38
	Jinjusi	250	186
	Changnyeonggun	101	83
	ChangwonsiMasanHappogu	119	91
	ChangwonsiMasanHoewongu	144	112
	ChangwonsiSeongsangu	75	65
	ChangwonsiUichanggu	147	128
	ChangwonsiJinhaegu	85	70
	Tongyeongsi	114	87
	Hadonggun	57	41
	Hamangun	67	50
	Hamyanggun	40	28
	Hapcheongun	75	57
Jeju		486	380
	Seogwiposi	158	119
	Jejusi	328	261

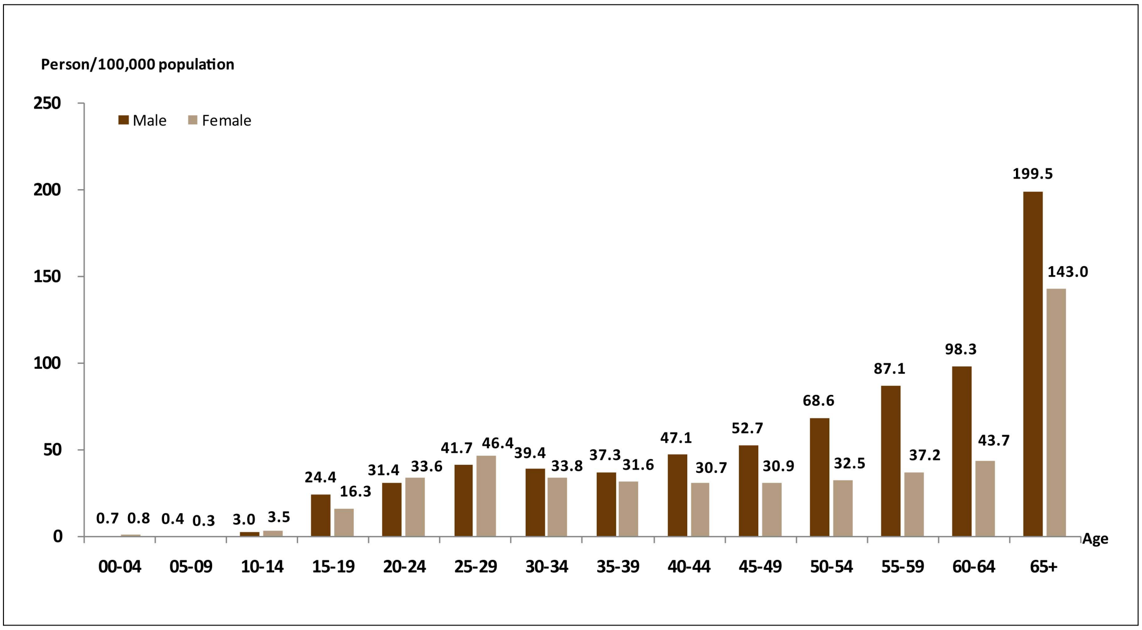

[Figure 3] (New) TB notification rates by gender and age, 2017

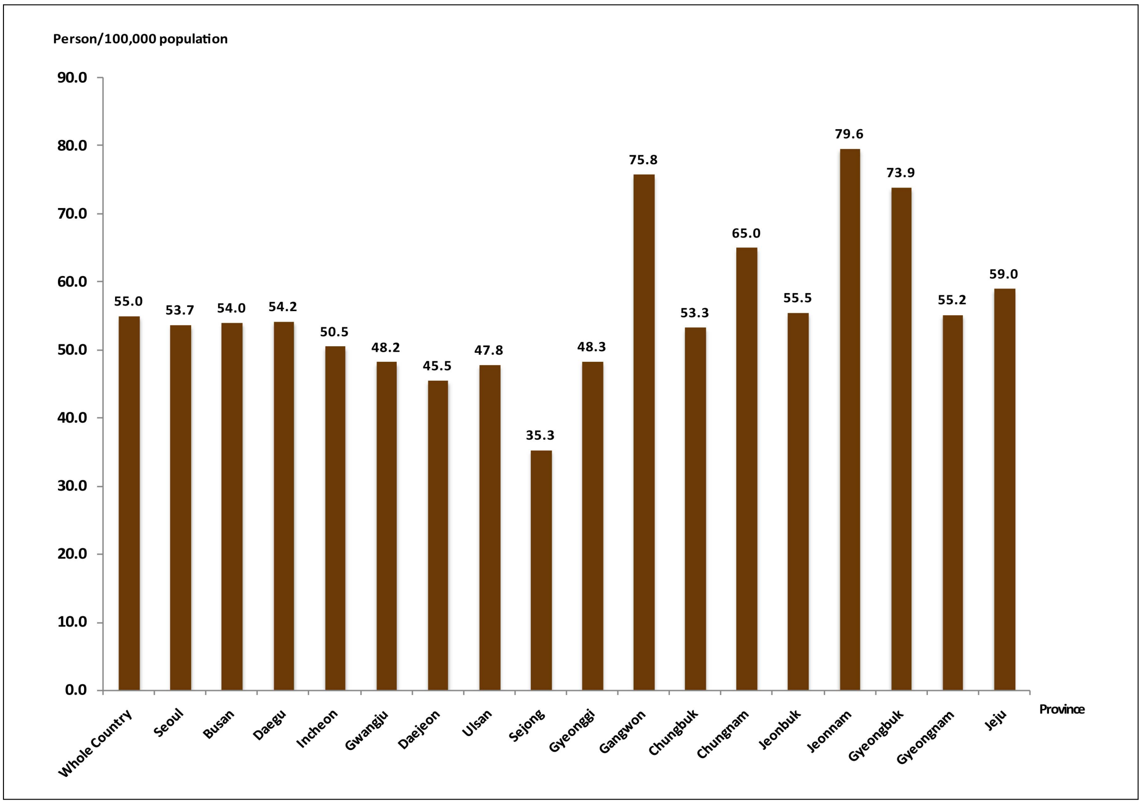

[Figure 4] (New) TB notification rates by province, 2017

Ⅳ. New Tuberculosis case notifications, 2001-2017

[Table 5] New TB notification cases and rates by gender and age, 2001-2017

Year	Gender \ Age	Total	0-4	5-9	10-14	15-19	20-24	25-29	30-34	35-39
2001*	Total	34,123	113	103	331	2,544	3,949	4,165	3,193	2,476
	Male	21,285	60	56	145	1,614	1,973	2,413	1,976	1,670
	Female	12,838	53	47	186	930	1,976	1,752	1,217	806
2002*	Total	32,010	84	80	232	2,113	3,544	3,736	3,010	2,328
	Male	19,806	50	39	117	1,325	1,735	2,141	1,822	1,575
	Female	12,204	34	41	115	788	1,809	1,595	1,188	753
2003*	Total	30,687	102	98	242	2,009	3,248	3,542	2,870	2,230
	Male	18,760	52	55	110	1,254	1,538	2,013	1,737	1,443
	Female	11,927	50	43	132	755	1,710	1,529	1,133	787
2004*	Total	31,503	79	41	208	2,009	2,969	3,505	2,983	2,366
	Male	19,172	49	26	88	1,242	1,415	1,955	1,765	1,513
	Female	12,331	30	15	120	767	1,554	1,550	1,218	853
2005	Total	35,269	123	79	251	2,007	3,038	3,789	3,100	2,631
		(73.0)	(4.8)	(2.4)	(7.2)	(65.2)	(79.3)	(98.1)	(71.1)	(62.1)
	Male	21,081	66	39	127	1,253	1,448	2,065	1,807	1,655
		(86.6)	(5.0)	(2.3)	(6.9)	(77.3)	(72.7)	(103.4)	(80.6)	(76.1)
	Female	14,188	57	40	124	754	1,590	1,724	1,293	976
		(59.2)	(4.7)	(2.6)	(7.6)	(51.8)	(86.4)	(92.5)	(61.1)	(47.4)
2006	Total	35,361	67	80	237	2,300	2,789	3,800	2,926	2,670
		(73.2)	(2.8)	(2.6)	(6.8)	(73.2)	(78.2)	(97.6)	(70.2)	(62.1)
	Male	20,918	31	46	115	1,408	1,344	2,076	1,666	1,623
		(86.2)	(2.5)	(2.8)	(6.2)	(85.2)	(72.7)	(103.5)	(77.7)	(74.0)
	Female	14,443	36	34	122	892	1,445	1,724	1,260	1,047
		(60.1)	(3.1)	(2.3)	(7.5)	(59.9)	(84.1)	(91.4)	(62.2)	(49.7)

* Only new patients were analyzed from previous annual report published from 2001 to 2004.

(Unit : person (person/100,000 population)) – continued –

40–44	45–49	50–54	55–59	60–64	65–69	70–74	75–79	80+
2,661	2,164	1,869	1,817	2,191	2,133	1,824	1,417	1,173
1,885	1,606	1,397	1,320	1,523	1,385	1,009	713	540
776	558	472	497	668	748	815	704	633
2,466	2,222	1,791	1,651	2,175	2,125	1,741	1,413	1,299
1,737	1,621	1,335	1,188	1,519	1,324	934	733	611
729	601	456	463	656	801	807	680	688
2,400	2,205	1,698	1,598	1,955	2,033	1,781	1,349	1,327
1,703	1,583	1,211	1,130	1,384	1,265	1,006	672	604
697	622	487	468	571	768	775	677	723
2,452	2,310	1,789	1,715	1,988	2,104	1,956	1,547	1,482
1,752	1,656	1,356	1,219	1,346	1,341	1,046	751	652
700	654	433	496	642	763	910	796	830
2,605<br>(61.5)	2,776<br>(69.9)	2,165<br>(75.2)	1,940<br>(84.0)	2,160<br>(112.9)	2,472<br>(145.7)	2,356<br>(189.6)	1,854<br>(242.0)	1,923<br>(283.7)
1,778<br>(82.4)	1,983<br>(98.8)	1,533<br>(106.0)	1,375<br>(119.4)	1,439<br>(157.2)	1,513<br>(196.2)	1,278<br>(249.1)	870<br>(316.8)	852<br>(424.6)
827<br>(39.8)	793<br>(40.4)	632<br>(44.1)	565<br>(48.7)	721<br>(72.2)	959<br>(103.6)	1,078<br>(147.8)	984<br>(200.3)	1,071<br>(224.4)
2,472<br>(59.0)	2,796<br>(68.2)	2,362<br>(76.5)	1,989<br>(84.9)	2,042<br>(106.1)	2,479<br>(143.2)	2,506<br>(190.0)	1,911<br>(235.0)	1,935<br>(267.9)
1,699<br>(79.2)	1,926<br>(92.7)	1,660<br>(106.7)	1,365<br>(117.1)	1,331<br>(145.0)	1,540<br>(196.3)	1,343<br>(245.3)	905<br>(311.3)	840<br>(395.6)
773<br>(37.9)	870<br>(43.0)	702<br>(45.8)	624<br>(52.9)	711<br>(70.6)	939<br>(99.2)	1,163<br>(150.7)	1,006<br>(192.5)	1,095<br>(214.8)

[Table 5] New TB notification cases and rates by gender and age, 2001–2017

Year	Gender \ Age	Total	0–4	5–9	10–14	15–19	20–24	25–29	30–34	35–39
2007	Total	34,710	61	63	250	2,361	2,567	3,520	2,743	2,668
		(71.6)	(2.6)	(2.1)	(7.3)	(73.8)	(75.8)	(89.3)	(68.2)	(61.2)
	Male	20,277	42	25	118	1,397	1,223	1,887	1,478	1,643
		(83.3)	(3.5)	(1.6)	(6.5)	(82.7)	(69.5)	(92.8)	(71.4)	(73.8)
	Female	14,433	19	38	132	964	1,344	1,633	1,265	1,025
		(59.9)	(1.7)	(2.7)	(8.2)	(63.9)	(82.6)	(85.5)	(64.8)	(48.0)
2008	Total	34,157	43	52	226	2,030	2,353	3,359	2,512	2,562
		(70.3)	(1.9)	(1.5)	(6.9)	(62.7)	(59.6)	(86.2)	(57.3)	(62.3)
	Male	19,877	24	30	108	1,231	1,106	1,733	1,456	1,541
		(81.4)	(2.1)	(1.7)	(6.2)	(73.0)	(54.4)	(86.3)	(64.9)	(73.2)
	Female	14,280	19	22	118	799	1,247	1,626	1,056	1,021
		(59.0)	(1.8)	(1.4)	(7.7)	(51.5)	(65.2)	(86.0)	(49.3)	(50.9)
2009	Total	35,845	46	51	251	2,170	2,419	3,303	2,560	2,629
		(73.5)	(2.1)	(1.9)	(7.7)	(64.8)	(76.9)	(85.4)	(66.9)	(60.3)
	Male	20,547	25	27	120	1,360	1,150	1,729	1,369	1,549
		(83.9)	(2.2)	(1.9)	(7.0)	(76.4)	(69.8)	(86.6)	(69.3)	(69.3)
	Female	15,298	21	24	131	810	1,269	1,574	1,191	1,080
		(63.0)	(2.0)	(1.9)	(8.4)	(51.6)	(84.7)	(84.0)	(64.3)	(50.9)
2010	Total	36,305	41	51	267	2,071	2,369	2,905	2,542	2,548
		(74.3)	(1.9)	(2.0)	(8.4)	(60.9)	(76.1)	(78.1)	(66.4)	(59.7)
	Male	20,555	22	24	138	1,318	1,187	1,504	1,371	1,500
		(83.8)	(1.9)	(1.8)	(8.3)	(72.9)	(72.3)	(78.3)	(69.3)	(68.4)
	Female	15,750	19	27	129	753	1,182	1,401	1,171	1,048
		(64.7)	(1.8)	(2.2)	(8.5)	(47.2)	(80.3)	(77.9)	(63.3)	(50.5)
2011	Total	39,557	59	38	305	2,030	2,496	3,127	2,852	2,620
		(78.9)	(2.6)	(1.6)	(9.6)	(57.3)	(79.0)	(86.9)	(72.2)	(61.3)
	Male	22,371	30	24	132	1,285	1,241	1,577	1,528	1,526
		(89.2)	(2.5)	(1.9)	(7.9)	(68.3)	(74.7)	(85.1)	(75.8)	(70.1)
	Female	17,186	29	14	173	745	1,255	1,550	1,324	1,094
		(68.7)	(2.6)	(1.2)	(11.3)	(44.8)	(83.7)	(88.7)	(68.4)	(52.2)
2012[1)]	Total	39,545	59	42	199	1,630	2,365	2,895	2,659	2,422
		(78.5)	(2.5)	(1.8)	(6.5)	(46.5)	(72.7)	(85.4)	(66.3)	(58.7)
	Male	22,705	35	23	96	1,027	1,212	1,490	1,439	1,447
		(90.1)	(2.9)	(1.9)	(6.1)	(55.2)	(70.7)	(85.2)	(70.3)	(69.1)
	Female	16,840	24	19	103	603	1,153	1,405	1,220	975
		(66.9)	(2.1)	(1.7)	(7.1)	(36.6)	(74.9)	(85.6)	(62.2)	(48.1)

1) 32 cases with age unknown (Male 21 cases, Female 11 cases)

(Unit : person (person/100,000 population)) – continued –

40–44	45–49	50–54	55–59	60–64	65–69	70–74	75–79	80+
2,417 (58.5)	2,855 (68.0)	2,362 (71.5)	2,068 (85.6)	1,892 (97.0)	2,513 (140.9)	2,428 (175.8)	1,902 (217.8)	2,040 (264.2)
1,609 (75.9)	1,986 (93.5)	1,628 (97.8)	1,398 (116.3)	1,234 (131.8)	1,575 (193.8)	1,299 (223.5)	927 (293.0)	808 (353.6)
808 (40.1)	869 (41.8)	734 (44.8)	670 (55.2)	658 (64.9)	938 (96.5)	1,129 (141.2)	975 (175.1)	1,232 (226.7)
2,442 (57.5)	2,707 (76.9)	2,540 (101.7)	2,050 (72.2)	1,874 (93.1)	2,501 (137.7)	2,552 (177.7)	2,135 (228.1)	2,219 (268.4)
1,634 (76.1)	1,859 (104.6)	1,740 (140.1)	1,402 (94.6)	1,233 (126.6)	1,458 (175.8)	1,378 (225.7)	1,022 (295.8)	922 (373.9)
808 (38.5)	848 (48.6)	800 (63.7)	648 (47.8)	641 (61.7)	1,043 (105.7)	1,174 (142.2)	1,113 (188.4)	1,297 (223.5)
2,638 (63.8)	2,744 (64.7)	2,764 (74.0)	2,143 (81.9)	2,007 (95.5)	2,597 (142.7)	2,756 (185.5)	2,332 (233.2)	2,435 (274.5)
1,699 (80.6)	1,876 (87.5)	1,869 (99.3)	1,441 (110.7)	1,295 (126.6)	1,539 (184.7)	1,462 (229.7)	1,065 (282.6)	972 (365.1)
939 (46.4)	868 (41.4)	895 (48.4)	702 (53.3)	712 (66.1)	1,058 (107.2)	1,294 (152.4)	1,267 (203.3)	1,463 (235.6)
2,501 (59.8)	2,844 (67.8)	2,901 (74.2)	2,398 (85.5)	2,135 (97.6)	2,549 (140.7)	2,925 (191.6)	2,574 (241.2)	2,684 (282.0)
1,554 (72.9)	1,833 (86.2)	1,903 (96.8)	1,567 (112.3)	1,361 (127.5)	1,484 (178.0)	1,532 (232.6)	1,206 (293.7)	1,051 (365.8)
947 (46.1)	1,011 (48.9)	998 (51.4)	831 (58.9)	774 (69.2)	1,065 (108.9)	1,393 (160.4)	1,368 (208.4)	1,633 (245.8)
2,812 (62.6)	2,915 (69.1)	3,320 (80.2)	2,761 (91.0)	2,363 (104.3)	2,603 (138.1)	3,130 (196.7)	2,926 (270.8)	3,200 (330.3)
1,723 (75.6)	1,904 (88.3)	2,189 (105.0)	1,815 (120.4)	1,517 (136.9)	1,550 (177.0)	1,678 (244.3)	1,391 (339.6)	1,261 (452.4)
1,089 (49.2)	1,011 (49.1)	1,131 (55.0)	946 (61.9)	846 (73.1)	1,053 (104.3)	1,452 (160.6)	1,535 (228.8)	1,939 (281.0)
2,785 (61.0)	2,907 (70.1)	3,324 (77.5)	2,976 (91.6)	2,452 (104.8)	2,505 (133.4)	3,394 (200.6)	3,263 (282.9)	3,636 (350.6)
1,738 (75.0)	1,992 (93.9)	2,288 (105.9)	2,010 (124.4)	1,596 (139.3)	1,482 (168.5)	1,826 (247.4)	1,560 (350.3)	1,423 (473.5)
1,047 (46.6)	915 (45.1)	1,036 (48.7)	966 (59.2)	856 (71.6)	1,023 (102.6)	1,568 (164.3)	1,703 (240.6)	2,213 (300.4)

[Table 5] New TB notification cases and rates by gender and age, 2001–2017

Year	Gender \ Age	Total	0–4	5–9	10–14	15–19	20–24	25–29	30–34	35–39
2013[2)]	Total	36,089	40	25	155	1,281	2,099	2,465	2,442	2,070
		(71.4)	(1.7)	(1.1)	(5.4)	(37.2)	(62.7)	(76.4)	(60.4)	(52.1)
	Male	20,665	23	8	72	770	1,125	1,296	1,292	1,194
		(81.7)	(1.9)	(0.7)	(4.8)	(42.3)	(63.5)	(77.7)	(62.6)	(59.1)
	Female	15,424	17	17	83	511	974	1,169	1,150	876
		(61.0)	(1.5)	(1.5)	(6.0)	(31.4)	(61.7)	(75.0)	(58.1)	(44.8)
2014	Total	34,869	40	21	115	1,131	1,988	2,218	2,092	1,935
		(68.7)	(1.7)	(0.9)	(4.2)	(33.6)	(57.9)	(70.4)	(52.7)	(49.9)
	Male	19,974	25	12	46	710	1,075	1,135	1,130	1,091
		(78.7)	(2.1)	(1.0)	(3.2)	(40.2)	(59.0)	(69.4)	(55.7)	(55.3)
	Female	14,895	15	9	69	421	913	1,083	962	844
		(58.7)	(1.3)	(0.8)	(5.3)	(26.4)	(56.6)	(71.4)	(49.5)	(44.4)
2015	Total	32,181	23	16	128	1,014	1,671	1,928	1,794	1,689
		(63.2)	(1.0)	(0.7)	(5.0)	(30.9)	(47.6)	(61.6)	(47.3)	(43.5)
	Male	18,695	17	11	76	630	907	1,029	958	963
		(73.4)	(1.5)	(0.9)	(5.7)	(36.7)	(48.7)	(63.1)	(49.3)	(48.8)
	Female	13,486	6	5	52	384	764	899	836	726
		(52.9)	(0.5)	(0.4)	(4.2)	(24.5)	(46.4)	(59.9)	(45.1)	(38.1)
2016	Total	30,892	17	13	102	750	1,419	1,760	1,548	1,533
		(60.4)	(0.8)	(0.6)	(4.2)	(23.5)	(40.1)	(55.7)	(43.0)	(38.9)
	Male	17,865	8	7	41	463	698	927	841	860
		(70.0)	(0.7)	(0.6)	(3.3)	(27.8)	(37.2)	(56.1)	(45.6)	(42.9)
	Female	13,027	9	6	61	287	721	833	707	673
		(50.9)	(0.8)	(0.5)	(5.3)	(18.8)	(43.4)	(55.2)	(40.3)	(34.8)
2017	Total	28,161	16	8	75	622	1,135	1,429	1,244	1,379
		(55.0)	(0.7)	(0.3)	(3.2)	(20.5)	(32.4)	(43.9)	(36.7)	(34.5)
	Male	16,147	8	5	36	386	581	711	686	759
		(63.1)	(0.7)	(0.4)	(3.0)	(24.4)	(31.4)	(41.7)	(39.4)	(37.3)
	Female	12,014	8	3	39	236	554	718	558	620
		(46.8)	(0.8)	(0.3)	(3.5)	(16.3)	(33.6)	(46.4)	(33.8)	(31.6)

2) 5 cases with age unknown (Male 3 cases, Female 2 cases)

[Note] 1. Notified (new) TB rate before 2010 is based on the estimate while new case report rate after 2011 is based on the mid-year population estimates. Therefore, the case notification rate before 2010 (table 5) might be different from case notification rate in the annual report.

(Unit : person (person/100,000 population))

40-44	45-49	50-54	55-59	60-64	65-69	70-74	75-79	80+
2,608 (57.2)	2,650 (63.2)	3,107 (71.6)	2,758 (79.9)	2,227 (91.9)	2,240 (116.7)	3,060 (172.7)	3,008 (246.1)	3,849 (345.0)
1,603 (69.1)	1,783 (83.6)	2,082 (95.2)	1,860 (108.0)	1,463 (123.3)	1,403 (154.5)	1,717 (220.4)	1,440 (300.3)	1,531 (467.6)
1,005 (44.9)	867 (42.0)	1,025 (47.7)	898 (52.0)	764 (61.8)	837 (82.7)	1,343 (135.2)	1,568 (211.1)	2,318 (294.0)
2,450 (54.3)	2,546 (59.2)	2,978 (68.9)	2,916 (79.3)	2,228 (88.4)	2,163 (107.7)	2,971 (166.4)	3,131 (241.5)	3,946 (327.0)
1,499 (65.3)	1,698 (77.9)	2,033 (93.1)	1,962 (106.8)	1,485 (120.1)	1,334 (139.2)	1,650 (209.0)	1,484 (287.6)	1,605 (446.1)
951 (43.0)	848 (40.0)	945 (44.2)	954 (51.8)	743 (57.8)	829 (78.9)	1,321 (132.7)	1,647 (211.1)	2,341 (276.4)
2,078 (47.1)	2,211 (50.6)	2,699 (63.3)	2,785 (71.7)	2,207 (81.3)	2,069 (98.1)	2,665 (149.9)	3,071 (226.9)	4,133 (315.0)
1,287 (57.3)	1,539 (69.5)	1,903 (88.1)	1,911 (98.6)	1,515 (113.8)	1,289 (127.5)	1,490 (188.1)	1,483 (271.7)	1,687 (423.4)
791 (36.5)	672 (31.1)	796 (37.8)	874 (44.9)	692 (50.0)	780 (71.0)	1,175 (119.2)	1,588 (196.6)	2,446 (267.7)
1,863 (43.8)	2,165 (48.5)	2,448 (58.7)	2,719 (66.7)	2,227 (75.1)	2,176 (99.6)	2,402 (136.1)	3,057 (217.1)	4,693 (330.1)
1,198 (55.4)	1,493 (66.2)	1,719 (81.0)	1,940 (95.4)	1,556 (107.0)	1,404 (133.9)	1,346 (169.4)	1,471 (255.9)	1,893 (430.4)
665 (31.8)	672 (30.5)	729 (35.5)	779 (38.2)	671 (44.4)	772 (67.9)	1,056 (108.8)	1,586 (190.3)	2,800 (285.2)
1,602 (39.0)	1,902 (41.9)	2,088 (50.9)	2,623 (62.1)	2,240 (70.5)	1,972 (87.2)	2,196 (124.4)	2,919 (193.8)	4,711 (307.5)
981 (47.1)	1,210 (52.7)	1,433 (68.6)	1,837 (87.1)	1,534 (98.3)	1,271 (116.9)	1,268 (157.8)	1,507 (241.8)	1,934 (400.7)
621 (30.7)	692 (30.9)	655 (32.5)	786 (37.2)	706 (43.7)	701 (59.7)	928 (96.4)	1,412 (159.9)	2,777 (264.7)

[Table 6] New TB notification cases and rates by regions, 2005–2017

Year	Whole Country	Seoul	Busan	Daegu	Incheon	Gwangju	Daejeon	Ulsan
2005	35,269 (72.4)	8,100 (79.6)	3,173 (86.9)	1,421 (56.4)	1,593 (61.5)	943 (67.3)	691 (47.7)	545 (50.3)
2006	35,361 (72.3)	7,617 (74.9)	3,499 (96.5)	1,293 (51.6)	1,512 (57.9)	745 (53.0)	692 (47.4)	587 (53.8)
2007	34,710 (70.6)	7,745 (76.0)	3,484 (96.8)	1,512 (60.6)	1,639 (62.0)	772 (54.7)	609 (41.4)	793 (72.3)
2008	34,157 (69.1)	7,252 (71.1)	3,202 (89.5)	1,661 (66.6)	1,541 (57.5)	768 (54.2)	913 (61.8)	798 (72.1)
2009	35,845 (72.2)	7,499 (73.5)	3,027 (85.2)	1,973 (79.2)	1,757 (65.0)	905 (63.4)	1,047 (70.6)	780 (70.0)
2010	36,305 (72.8)	7,534 (74.0)	3,274 (92.7)	1,999 (80.3)	1,897 (69.8)	834 (58.0)	995 (66.9)	870 (77.9)
2011	39,557 (78.9)	8,028 (79.4)	3,357 (95.5)	2,211 (88.9)	1,997 (72.6)	948 (65.4)	959 (64.0)	966 (86.0)
2012	39,545 (78.5)	8,261 (82.1)	3,278 (93.6)	2,150 (86.5)	1,983 (71.0)	1,072 (73.6)	884 (58.6)	792 (69.8)
2013	36,089 (71.4)	7,389 (73.8)	2,766 (79.2)	1,882 (75.8)	1,908 (67.3)	887 (60.7)	829 (54.6)	820 (71.6)
2014	34,869 (68.7)	6,831 (68.5)	2,548 (73.1)	1,881 (75.9)	1,766 (61.7)	817 (55.8)	859 (56.5)	738 (63.9)
2015	32,181 (63.2)	6,011 (60.7)	2,386 (68.6)	1,518 (61.4)	1,646 (57.0)	803 (54.8)	730 (48.2)	634 (54.5)
2016	30,892 (60.4)	5,769 (58.8)	2,221 (64.1)	1,466 (59.4)	1,635 (56.3)	737 (50.4)	677 (45.0)	651 (55.8)
2017	28,161 (55.0)	5,226 (53.7)	1,861 (54.0)	1,333 (54.2)	1,472 (50.5)	703 (48.2)	681 (45.5)	556 (47.8)

[Note] 1. Sejong city was established on July 2012 as Sejong city was separated from Chungchungnam-do.

(Unit : person (person/100,000 population))

Sejong	Gyeonggi	Gangwon	Chungbuk	Chungnam	Jeonbuk	Jeonnam	Gyeongbuk	Gyeongnam	Jeju
–	6,275	1,657	1,056	1,564	1,709	1,631	1,978	2,595	338
	(59.3)	(109.2)	(70.9)	(79.9)	(90.1)	(82.5)	(73.5)	(82.3)	(60.7)
–	6,237	1,769	1,023	1,654	1,606	1,671	2,099	3,007	350
	(57.7)	(117.2)	(68.6)	(84.0)	(85.6)	(85.5)	(78.1)	(95.0)	(62.7)
–	6,146	1,474	925	1,588	1,452	1,526	2,091	2,691	263
	(55.8)	(98.0)	(61.6)	(80.0)	(77.8)	(78.8)	(77.9)	(84.5)	(47.1)
–	5,884	1,442	981	1,643	1,447	1,414	2,158	2,618	435
	(52.5)	(95.7)	(64.8)	(81.9)	(77.8)	(73.5)	(80.6)	(81.5)	(77.7)
–	5,978	1,536	1,039	1,624	1,660	1,642	2,459	2,504	415
	(52.5)	(101.7)	(68.2)	(80.1)	(89.5)	(85.7)	(92.0)	(77.3)	(73.9)
–	6,141	1,469	1,002	1,577	1,491	1,514	2,522	2,719	467
	(53.1)	(97.0)	(65.4)	(77.0)	(80.4)	(79.3)	(94.5)	(83.4)	(82.8)
–	7,761	1,420	1,126	1,492	1,534	1,700	2,970	2,610	478
	(66.0)	(93.5)	(72.9)	(72.0)	(82.5)	(89.4)	(111.0)	(79.7)	(84.1)
72	7,818	1,413	1,187	1,476	1,345	1,942	2,789	2,585	498
(69.6)	(65.6)	(92.7)	(76.6)	(73.5)	(72.3)	(102.3)	(104.0)	(78.5)	(86.6)
72	7,523	1,371	1,018	1,374	1,224	1,677	2,536	2,405	408
(61.7)	(62.4)	(89.8)	(65.3)	(67.9)	(65.8)	(88.5)	(94.6)	(72.8)	(69.9)
71	7,177	1,555	998	1,506	1,304	1,676	2,472	2,295	375
(51.3)	(58.8)	(101.6)	(63.7)	(73.8)	(70.1)	(88.5)	(92.2)	(69.1)	(62.9)
80	6,782	1,368	986	1,455	1,206	1,657	2,328	2,217	374
(43.8)	(55.0)	(89.2)	(62.8)	(70.8)	(64.9)	(87.5)	(86.7)	(66.4)	(61.2)
103	6,577	1,298	977	1,483	1,281	1,515	2,214	1,977	311
(45.6)	(52.6)	(84.5)	(61.9)	(71.5)	(69.1)	(80.0)	(82.5)	(59.0)	(49.5)
92	6,130	1,165	844	1,359	1,025	1,503	1,978	1,853	380
(35.3)	(48.3)	(75.8)	(53.3)	(65.0)	(55.5)	(79.6)	(73.9)	(55.2)	(59.0)

[Table 7] New TB notification cases and rates by pathological location and result of sputum smear test, 2005–2017

(Unit : person (person/100,000 population))

Year	Total	Pulmonary TB		Extra Pulmonary TB							
			Smear Positive	Sub-Total	Pleural	Lymphatic	Abdominal	Bone-Joint	CNS	Genitourinary	Other
2005	35,269	30,098	11,638	5,171	1,568	1,009	490	290	153	130	1,531
	(72.4)	(61.8)	(23.9)	(10.6)							
2006	35,361	30,317	11,513	5,044	1,409	964	448	242	161	120	1,700
	(72.3)	(62.0)	(23.6)	(10.3)							
2007	34,710	29,705	10,927	5,005	1,409	901	477	208	129	137	1,744
	(70.6)	(60.5)	(22.2)	(10.2)							
2008	34,157	28,344	11,048	5,813	1,545	1,037	565	275	196	123	2,072
	(69.1)	(57.4)	(22.4)	(11.8)							
2009	35,845	28,922	11,285	6,923	1,979	1,326	721	274	205	146	2,272
	(72.2)	(58.2)	(22.7)	(13.9)							
2010	36,305	28,176	10,776	8,129	2,569	1,771	829	373	290	168	2,129
	(72.8)	(56.5)	(21.6)	(16.3)							
2011	39,557	30,100	11,714	9,457	3,167	1,993	1,155	520	337	240	2,045
	(78.9)	(60.1)	(23.4)	(18.9)							
2012	39,545	31,075	12,137	8,470	2,884	1,752	1,184	549	337	237	1,582
	(78.5)	(61.7)	(24.1)	(16.8)							
2013	36,089	28,720	11,100	7,369	2,275	1,832	1,172	447	345	213	1172
	(71.4)	(56.8)	(22.0)	(14.6)							
2014	34,869	27,906	10,446	6,963	2,568	1,780	1,133	418	288	192	636
	(68.7)	(55.0)	(20.6)	(13.7)							
2015	32,181	25,550	9,309	6,631	2,685	1,526	1,041	456	323	173	473
	(63.2)	(50.1)	(18.3)	(13.0)							
2016	30,892	24,696	8,812	6,196	2,492	1,362	1,040	380	265	192	512
	(60.4)	(48.3)	(17.2)	(12.1)							
2017	28,161	22,314	7,701	5,847	2,237	1,176	949	363	310	169	591
	(55.0)	(43.6)	(15.0)	(11.4)							

[Notese] 1. If a patient was notified as both pulmonary TB and extra pulmonary TB case, the case was classified into the reported pulmonary TB case only.
2. There are duplicated report cases in terms of pathologic locations in case of extra pulmonary TB. So, there may be difference between subtotal of extra pulmonary TB cases and the number of extra pulmonary TB cases based on pathologic locations.
3. Calculation errors of the number extra pulmonary TB cases based on pathologic locations in from 2001 to 2013 is corrected in this annual report.

[Table 8] New TB notification cases and rates by type of healthcare provider, 2001–2017

(Unit : person (%)[1])

Year	otal	Public health center	Private medical facilities			
			Sub–Total	General Hospital	Hospital	Clinic
2001	34,123 (100.0)	15,728 (46.1)	18,395 (53.9)	11,724 (34.4)	1,848 (5.4)	4,823 (14.1)
2002	32,010 (100.0)	13,003 (40.6)	19,007 (59.4)	11,931 (37.3)	2,296 (7.2)	4,780 (14.9)
2003	30,687 (100.0)	11,810 (38.5)	18,877 (61.5)	11,966 (39.0)	2,531 (8.2)	4,380 (14.3)
2004	31,503 (100.0)	10,851 (34.4)	20,652 (65.6)	13,110 (41.6)	3,112 (9.9)	4,430 (14.1)
2005	35,269 (100.0)	9,680 (27.4)	25,589 (72.6)	16,454 (46.7)	3,646 (10.3)	5,489 (15.6)
2006	35,361 (100.0)	9,018 (25.5)	26,343 (74.5)	17,739 (50.2)	3,697 (10.5)	4,907 (13.9)
2007	34,710 (100.0)	7,558 (21.8)	27,152 (78.2)	19,606 (56.5)	3,298 (9.5)	4,248 (12.2)
2008	34,157 (100.0)	7,315 (21.4)	26,842 (78.6)	19,981 (58.5)	3,141 (9.2)	3,720 (10.9)
2009	35,845 (100.0)	7,079 (19.7)	28,766 (80.3)	22,633 (63.1)	3,036 (8.5)	3,097 (8.6)
2010	36,305 (100.0)	5,463 (15.0)	30,842 (85.0)	25,611 (70.5)	2,871 (7.9)	2,360 (6.5)
2011	39,557 (100.0)	4,461 (11.3)	35,096 (88.7)	29,207 (73.8)	3,704 (9.4)	2,185 (5.5)
2012	39,545 (100.0)	3,779 (9.6)	35,766 (90.4)	29,335 (74.2)	4,121 (10.4)	2,310 (5.8)
2013	36,089 (100.0)	3,269 (9.1)	32,820 (90.9)	27,565 (76.4)	3,743 (10.4)	1,512 (4.2)
2014	34,869 (100.0)	2,994 (8.6)	31,875 (91.4)	27,502 (78.9)	3,310 (9.5)	1,063 (3.0)
2015	32,181 (100.0)	2,320 (7.2)	29,861 (92.8)	25,919 (80.5)	3,012 (9.4)	930 (2.9)
2016	30,892 (100.0)	2,405 (7.8)	28,487 (92.2)	25,175 (81.5)	2,556 (8.3)	756 (2.4)
2017	28,161 (100.0)	1,516 (5.4)	26,645 (94.6)	23,730 (84.3)	2,282 (8.1)	633 (2.2)

1) Percentage based on total.

[Table 9] New TB notification cases in foreign born, 2001–2017

(Unit : person)

Year	Total cases	New cases			
		Sub-Total	Pulmonary TB (Smear Positive)[1]		Extra Pulmonary TB
2001	152	126	112	(38)	14
2002	170	149	138	(45)	11
2003	228	188	176	(58)	12
2004	315	258	232	(71)	26
2005	388	312	263	(86)	49
2006	481	397	349	(123)	48
2007	588	489	405	(136)	84
2008	736	587	486	(167)	101
2009	637	519	414	(131)	105
2010[2]	849	678	546	(188)	132
2011	1,213	1,007	748	(248)	259
2012	1,510	1,227	956	(320)	271
2013	1,737	1,420	1,119	(339)	301
2014	1,858	1,566	1,262	(336)	304
2015	1,944	1,589	1,287	(398)	302
2016	2,569	2,123	1,810	(452)	313
2017	2,045	1,632	1,355	(353)	277

1) Smear-positive cases among pulmonary TB patients.
2) Calculation errors of New TB cases to foreigner in 2010 is corrected in this annual report.

Ⅴ. Multidrug-resistant tuberculosis case notifications, 2011-2017

[Table 10] Multidrug-resistant TB notification cases, 2011-2017

Year	Age / Gender	Total	0-4	5-9	10-14	15-19	20-24	25-29	30-34	35-39
2011	Total	975	0	0	0	23	54	96	106	77
	Male	701	0	0	0	14	24	51	61	56
	Female	274	0	0	0	9	30	45	45	21
2012[1)]	Total	1,212	0	0	1	34	74	102	94	113
	Male	818	0	0	0	17	40	51	59	79
	Female	394	0	0	1	17	34	51	35	34
2013[2)]	Total	951	0	0	3	16	68	81	89	74
	Male	626	0	0	1	11	30	35	40	46
	Female	325	0	0	2	5	38	46	49	28
2014	Total	856	0	1	2	20	49	80	89	62
	Male	575	0	1	1	11	29	38	53	37
	Female	281	0	0	1	9	20	42	36	25
2015	Total	787	0	0	0	16	67	78	75	54
	Male	538	0	0	0	12	38	42	42	36
	Female	249	0	0	0	4	29	36	33	18
2016	Total	852	0	0	2	17	52	69	68	56
	Male	552	0	0	0	10	31	38	41	35
	Female	300	0	0	2	7	21	31	27	21
2017	Total	689	0	1	0	11	30	39	48	58
	Male	464	0	1	0	6	18	23	32	42
	Female	225	0	0	0	5	12	16	16	16

1) 1 case with age unknown (Male)
2) 1 case with age unknown (Male)

※ Calculation by Patients with multidrug-resistant tuberculosis
- A patient who is reported to have multidrug-resistant tuberculosis in the year or is converted to multidrug-resistant TB among past cases.
- Patient with multidrug-resistant tuberculosis in the year = A∪B-C
  - A : A patient who is reported to have multidrug-resistant TB among the patient notified in 2017
  - B : A patient who was found to be multidrug-resistant in the test results found in drug-resistant examination data between 2016 and 2017
  - C : A patient who was reported to have multidrug-resistant TB in the past.
- The same analysis methods applies to the extensively drug-resistant TB as to multidrug-resistant TB.

※ Patients with multidrug resistance include those who have the extensively drug resistance(However, the patients who were filed as those who had the multidrug resistance in the past and were also filed as having the extensively drug resistance in the relevant year were included in the category of patients who have the extensively drug resistance in the relevant year only.)

(Unit : person)

40–44	45–49	50–54	55–59	60–64	65–69	70–74	75–79	80+
112	100	110	89	64	55	34	35	20
91	81	92	76	52	43	25	25	10
21	19	18	13	12	12	9	10	10
129	135	137	113	87	57	57	45	33
94	98	110	83	72	40	39	22	13
35	37	27	30	15	17	18	23	20
84	101	110	100	49	51	49	40	35
55	83	92	82	36	36	36	24	18
29	18	18	18	13	15	13	16	17
83	74	81	78	60	43	43	44	47
56	60	71	64	47	34	29	26	18
27	14	10	14	13	9	14	18	29
71	73	84	72	54	46	40	23	34
52	64	68	58	43	31	30	14	8
19	9	16	14	11	15	10	9	26
70	74	103	81	64	42	39	47	68
47	58	75	66	52	30	20	28	21
23	16	28	15	12	12	19	19	47
61	58	71	75	58	42	41	39	57
40	47	58	58	42	33	21	22	21
21	11	13	17	16	9	20	17	36

[Table 11] Extensively drug-resistant TB notification cases, 2011-2017

Year	Age / Gender	Total	0-4	5-9	10-14	15-19	20-24	25-29	30-34	35-39
2011	Total	140	0	0	0	3	5	5	13	8
	Male	101	0	0	0	2	2	3	4	6
	Female	39	0	0	0	1	3	2	9	2
2012	Total	158	0	0	0	4	7	15	13	12
	Male	101	0	0	0	3	5	8	5	7
	Female	57	0	0	0	1	2	7	8	5
2013	Total	113	0	0	0	0	9	14	8	10
	Male	69	0	0	0	0	3	5	4	7
	Female	44	0	0	0	0	6	9	4	3
2014	Total	63	0	0	0	0	2	13	5	2
	Male	38	0	0	0	0	1	7	2	2
	Female	25	0	0	0	0	1	6	3	0
2015	Total	58	0	0	0	0	5	7	4	6
	Male	41	0	0	0	0	3	4	2	4
	Female	17	0	0	0	0	2	3	2	2
2016	Total	59	0	0	0	2	2	10	7	7
	Male	42	0	0	0	2	0	7	4	6
	Female	17	0	0	0	0	2	3	3	1
2017	Total	55	0	0	0	1	2	3	3	7
	Male	40	0	0	0	0	1	1	3	6
	Female	15	0	0	0	1	1	2	0	1

※ The same analysis methods applies to the extensively drug-resistant TB as to multidrug-resistant TB.

(Unit : person)

40–44	45–49	50–54	55–59	60–64	65–69	70–74	75–79	80+
17	17	20	15	13	12	6	3	3
14	14	14	13	11	10	4	2	2
3	3	6	2	2	2	2	1	1
14	17	17	14	16	13	7	5	4
8	10	13	11	14	8	4	2	3
6	7	4	3	2	5	3	3	1
5	7	13	13	9	6	12	2	5
4	3	9	9	7	3	12	1	2
1	4	4	4	2	3	0	1	3
8	1	7	8	8	5	2	2	0
4	1	5	6	4	3	2	1	0
4	0	2	2	4	2	0	1	0
8	8	4	3	5	4	2	1	1
5	8	4	2	5	1	2	1	0
3	0	0	1	0	3	0	0	1
4	8	2	3	5	2	3	3	1
3	5	2	3	4	2	3	1	0
1	3	0	0	1	0	0	2	1
4	3	11	7	3	3	3	3	2
3	3	8	6	2	3	2	2	0
1	0	3	1	1	0	1	1	2

Ⅵ. APPENDIX

1. The number of deaths of tuberculosis by gender and age, 2001–2016

Year	Gender \ Age	Total	0–4	5–9	10–14	15–19	20–24	25–29	30–34	35–39
2001	Total	3,218	5	2	2	11	22	44	75	92
	Male	2,303	2	2	1	6	11	26	58	71
	Female	915	3	0	1	5	11	18	17	21
2002	Total	3,350	5	2	3	9	19	42	57	100
	Male	2,401	1	1	1	6	11	25	33	84
	Female	949	4	1	2	3	8	17	24	16
2003	Total	3,329	1	0	2	10	18	46	66	106
	Male	2,331	1	0	1	5	10	24	36	69
	Female	998	0	0	1	5	8	22	30	37
2004	Total	2,940	1	2	1	9	23	47	79	88
	Male	2,069	1	1	0	5	10	20	56	69
	Female	871	0	1	1	4	13	27	23	19
2005	Total	2,893	1	3	2	6	16	37	61	72
	Male	1,991	0	1	1	2	5	24	47	51
	Female	902	1	2	1	4	11	13	14	21
2006	Total	2,726	4	0	2	2	18	34	51	76
	Male	1,876	3	0	1	0	8	20	30	49
	Female	850	1	0	1	2	10	14	21	27
2007	Total	2,376	2	1	1	4	8	22	46	57
	Male	1,642	0	0	1	2	6	14	34	46
	Female	734	2	1	0	2	2	8	12	11
2008	Total	2,323	2	0	1	4	10	23	36	56
	Male	1,557	2	0	1	2	6	15	23	44
	Female	766	0	0	0	2	4	8	13	12
2009	Total	2,292	0	0	0	3	7	14	26	44
	Male	1,526	0	0	0	2	3	10	19	34
	Female	766	0	0	0	1	4	4	7	10
2010	Total	2,365	0	0	0	2	6	13	27	56
	Male	1,524	0	0	0	1	4	10	19	37
	Female	841	0	0	0	1	2	3	8	19

(Unit : person) − continued −

40–44	45–49	50–54	55–59	60–64	65–69	70–74	75–79	80+	unknown
218	187	183	220	292	338	390	485	652	0
176	149	158	184	244	286	285	304	340	0
42	38	25	36	48	52	105	181	312	0
192	195	176	218	302	363	409	467	791	0
158	165	146	184	257	281	295	304	449	0
34	30	30	34	45	82	114	163	342	0
179	189	209	200	254	355	444	447	803	0
149	156	176	170	216	281	319	285	433	0
30	33	33	30	38	74	125	162	370	0
155	186	161	186	223	295	323	424	737	0
133	156	141	150	185	241	234	273	394	0
22	30	20	36	38	54	89	151	343	0
136	209	152	171	194	288	359	436	750	0
107	181	129	155	157	239	247	270	375	0
29	28	23	16	37	49	112	166	375	0
114	167	183	165	178	248	370	383	731	0
96	139	166	130	141	206	265	244	378	0
18	28	17	35	37	42	105	139	353	0
111	149	137	128	150	236	299	343	682	0
100	120	115	109	125	187	212	227	344	0
11	29	22	19	25	49	87	116	338	0
77	134	117	124	113	212	322	350	742	0
67	112	103	107	84	158	229	211	393	0
10	22	14	17	29	54	93	139	349	0
78	127	139	92	119	251	292	370	729	1
63	113	124	77	104	196	214	222	345	0
15	14	15	15	15	55	78	148	384	1
68	100	141	107	141	177	279	362	886	0
59	82	115	93	115	141	196	228	424	0
9	18	26	14	26	36	83	134	462	0

1. The number of deaths of tuberculosis by gender and age, 2001–2016

Year	Age / Gender	Total	0–4	5–9	10–14	15–19	20–24	25–29	30–34	35–39
2011	Total	2,364	0	0	0	1	5	9	22	37
	Male	1,499	0	0	0	0	3	7	15	29
	Female	865	0	0	0	1	2	2	7	8
2012	Total	2,466	2	0	2	1	4	8	21	36
	Male	1,588	1	0	1	0	2	5	14	27
	Female	878	1	0	1	1	2	3	7	9
2013	Total	2,230	0	0	0	0	5	13	12	24
	Male	1,455	0	0	0	0	3	10	9	20
	Female	775	0	0	0	0	2	3	3	4
2014	Total	2,305	0	0	0	2	2	5	12	24
	Male	1,480	0	0	0	1	2	3	8	15
	Female	825	0	0	0	1	0	2	4	9
2015	Total	2,209	0	0	0	0	0	8	18	15
	Male	1,390	0	0	0	0	0	3	11	10
	Female	819	0	0	0	0	0	5	7	5
2016	Total	2,186	0	0	0	0	5	3	6	16
	Male	1,349	0	0	0	0	2	1	5	11
	Female	837	0	0	0	0	3	2	1	5

※ The number of deaths of tuberculosis by gender and age is sourced from “Annual Report on the Causes of Death Statistics” released on the end of every September by Statistics Korea. (The number of deaths of tuberculosis in 2017 is acquired from the report released by Statistics Korea at the end of September 2018.)

(Unit : person)

40–44	45–49	50–54	55–59	60–64	65–69	70–74	75–79	80+	unknown
53	88	130	127	126	150	294	416	906	0
46	75	116	112	106	120	194	260	416	0
7	13	14	15	20	30	100	156	490	0
54	80	114	114	107	154	312	484	971	2
46	69	99	100	89	116	228	322	467	2
8	11	15	14	18	38	84	162	504	0
43	81	112	92	119	152	261	382	934	0
42	76	101	75	95	116	201	261	446	0
1	5	11	17	24	36	60	121	488	0
40	69	117	110	104	144	293	385	998	0
33	62	106	90	80	108	221	248	503	0
7	7	11	20	24	36	72	137	495	0
37	66	113	117	98	141	201	371	1,023	1
32	56	92	101	86	119	134	242	503	1
5	10	21	16	12	22	67	129	520	0
27	64	87	95	97	120	177	369	1,120	0
22	51	74	81	83	97	132	232	558	0
5	13	13	14	14	23	45	137	562	0

2. Tuberculosis Case Notification · Reporting Form

■ Enforcement Regulations on Tuberculosis Prevention Act [Annex, Form 1] 〈Revision 2016.8.4.〉

TUBERCULOSIS CASE NOTIFICATION·REPORTING FORM

(1page/4page)

Receiver: ____________ The head of Public Health center FAX No.: ____________________

※ This notification·reporting form is used for notification of incidents or reporting the treatment results when TB patients are diagnosed or treated, when TB patients expired and when postmortem examinations were conducted on TB patient's body.
※ Mark the appropriate blank with √ mark or fill it in.

■ Notification: [ ]Diagnosis and treatment of TB patients
[ ] Death of TB patients and postmortem examination on TB patient's body (Date of expiration: _______)
([ ] Death caused by tuberculosis, [ ] death caused by other than tuberculosis))

A. Personal profile of the patient or the deceased

(1) Name: [ ]	(2) Korean ID No:
(3) Age:	(4) Gender: [ ]Male, [ ]Female
(5) Nationality(For foreigner only): [ ]	(6) Date of Entry(For foreigner only): DD MM YY
(7) Telephone No:	(8) Mobile:
(9) Address:	

※ (As the data in (10), (11) and (12) are the data required for epidemiological investigation, you shall fill them in.
(10) Occupation: [ ]School personnel, [ ]Health/Medical worker, [ ]Students, [ ]Soldier, [ ]Hairdresser, [ ]Food/restaurant business,
[ ]Sailor(the deep-sea), [ ]Stewardess /flight attendance, [ ]others( )

(11) Name of facilities (company or school, etc.):

(12) Address of facilities (company or school, etc.):

B. Information on examination, diagnosis and treatment

[Examination for Diagnosis] ※ Mark the appropriate blank with √ mark. (Fill in the date and type of samples other than sputum in (15) and (16).
※ The initial examination has to be conducted. If the examination has not been conducted or is under progress at the time of notification, the supplementary notification has to be made depending on the results of examination.

(13) Type of initial examination	(14) Status and results of examination					(15) Date specimen collected (or date of examination)	(16) Type of specimen
	Not conducted	Under examination	Completion of examination				
			Positive	Negative	Unknown		
Chest X-ray result			Suspected TB	Normal		DD MM YY	
Smear microscopy result						DD MM YY	[ ] sputum,[ ] others
Culture result				NTM included		DD MM YY	[ ] sputum,[ ] others
Nucleic acid amplification test (TB-PCR test)						DD MM YY	[ ] sputum,[ ] others
Xpert MTB/RIF tests						MM YY	[ ] sputum,[ ] others
Histologic exam results						DD MM YY	[ ]

[Diganosis and initial treatment drugs]

(17) International codes for diseases: [ ][ ][ ].[ ][ ]
※ Fill it in down to the narrow classification (to one place of decimal).

(18) Type of tuberculosis
[ ]Pulmonary TB (tuberculosis which invades into the lung parenchyme, larynx, trachea and bronchus, or military tuberulosis)
[ ]Extra pulmonary TB (Location of lesions:)
[ ]Pulmonary TB + Extra pulmonary TB (Location of lesions:)

(19) Classification of patient
[ ]New patient(initial treatment case)
[ ]Previously treated patient ([ ]Relapse,
[ ]Treatment after failure,
[ ]Treatment after loss to follow-up,
[ ]Other previously treated)
[ ]Patient with unknown previous TB treatment history

(20) Whether the treatment was conducted in the medical institute:
[ ] Date (or expected date) of starting the treatment: ______________
[ ] Not treated ※ It includes the case in which the patient is diagnosed in the medical institute but not treated and transferred to other institutes.

(21) Medication: [ ]H, [ ]R, [ ]E, [ ]Z, [ ]Rfb, [ ]Km, [ ]Amk, [ ] Cm, [ ]S, [ ]Lfx, [ ]Mfx, [ ]Ofx, [ ]Pto, [ ]Cs, [ ]PAS, [ ]Lzd, [ ]Clr, [ ]others( )

[Drug susceptibility test] ※ The result shall be notified whenever the drug susceptibility test is conducted.

(22) Result of drug susceptibility test: [ ] Not conducted, [ ] Under examination,
[ ]Test completed ([ ] No resistance found, [ ] Resistance found)

(23) Method of drug susceptibility test: [ ] traditional method, [ ] rapid detection of INH/RFP resistance,
[ ] Real time dual polymerase chain reaction test (Xpert MTB/RIF tests and etc.)

(24) TB resistant drug: [ ]H, [ ]R, [ ]E, [ ]Z, [ ]Rfb, [ ]Km, [ ]Amk, [ ] Cm, [ ]S, [ ]Lfx, [ ]Mfx, [ ]Ofx, [ ]Pto, [ ]Cs, [ ]PAS, [ ]Lzd, [ ]Clr, [ ]others( )

(25) Code of drug resistance TB: [ ]U88.0(Multidrug resistance TB), [ ]U88.1(Extenstively drug resistant TB),
[ ]Rifampin resistance TB

(26) Date specimen collected : ______________※ Date when the specimen for drug susceptibility test is collected from the patient
(Fill it when the examination is under way as well.)

■ Report of the treatment outcome

(27) Treatment outcome: [ ]Cured, [ ]Completed, [ ]Failed, [ ]Lost to follow-up, [ ]Died, [ ]Transfer-out,
[ ]Diagnosis changed([ ]NTM, [ ]Tumor, [ ]Other disease)

(28) Date of decision made on the treatment outcome
※ Date of last treatment when the patient is transferred to other medical institute

(29) Date of finishing the treatment: ______________

(30) Special to note:

[Notifier or reporter]

(31) Date of notification and reporting: ______________

(32) Code of care facility: [ ], name of care facility: [ ] Contact of care facilility: [ ]

(33) Name of physician: [ ], Permit no. of physician: [ ], Department of medicine: [ ] (signature or seal)

I notify or report the TB patient according to Article 8 of Tuberculosis Prevention Act and Article 3 of Enforcement Regulations of the Act.

210㎜×297㎜[Woodfree printing paper 80/㎡]

Description on the notifcation and report of TB patients

1. **Legal Regulations:** (Notification by physician) and Article 12 (Other mandatory reporter) of "Act on the Prevention and Control of Infectious Disease" and Article 8 (Report by Medical Institute) of "TB Preventoin Act"
2. **Notification and reporting period:** Immediate in the following cases
   A. Reporting
      1) When a physician diagnosed Tuberculosis(TB) or treated a TB patient or suspected TB patient.
      2) When a TB patient or suspected TB patient expired or a physician conducted a postmortem examination on a TB patient's body.
   B. Reporting: Results of treatment of TB patients or suspected TB patients notified according to the notification 1) above
3. **Subject to report:** TB patient and suspected TB patients (according to the diagnosis standard for the infectious disease (Article 6, Paragraph 4 of 'Enforcement Decree of Act on Prevention and Control of Infectious Diesase".)
   A. TB patient: an individual who has the clinical manifestations of TB and is found to have been infected by the pathogen in the following tests
      1) Smear-positive in the test with a patient specimen (sputum, blood, urine, CSF, tissues, etc.)
      2) Culture-positive for Mycobacterium Tuberculosis in the test with a patient specimen (sputum, blood, urine, CSF, tissues, etc.)
      3) Positive in the test of nucleic acid amplification with a patient specimen (sputum, blood, urine, CSF, tissues, etc.)
         * Especially, Mycobacterium bovis should be identified in the culture for confirmation of diagnosis.
   B. Suspected TB patient: An individual whose clinical, radiologic and histological presentations suggest TB infection but has yet to be confirmed bacteriologically to be infected by the pathogen.
4. **Methods of Case Notification**
   A. Where to notify: the head of public health center
   B. How to notify: fax and website (http://is.cdc.go.kr (Integrated disease and health control system - TB control, hereinafter called as 'electronic data control system')
   C. Form: according to 'Enforcement Regulations of Act on Prevention of TB'

How to notify

Required information: (2) Name, (3) Age, (4) Sex, (10) ~ (12) Job related information, (31) ~ (33) Information on reporter

1. **Notification**
   A. Personal information of patient and the deceased: Be sure to fill in here as the yellow blank in Page 1 of the form shall mandatorily be filled.
   B. Information on examination, diagnosis and treatment: Fill them all if they are available. If the items are not available as of notification as the information has not been conducted or the test was underway, the supplementary notification is possible depending on the result of the examination.
3. **Report of the treatment results:** The result of the treatment is reported when the medical institution finishes the treatment for the patients.

Classification and definition of tuberculosis patient (See Item (19) of the Form (Page 1))

Classification	Definition
New patient	A patient who has never been treated for Tuberculosis<br>※ who has taken anti-tuberculosis drugs for less than 1 month<br>※ who has received treatment in a hospital as a new case but has been transferred to other hospital without cure/completion/failure/default
Previously treated patient	A patient who has received 1 month or more of anti-TB drugs in past<br>※ They can be classified further by the outcom of their most recent course of treatment as follows
Relapse	A patient who has previously been treated for TB, were declared cured or treatment completed at the end of their most recent course of treatment, and are now diagnosed with a recurrent episode of TB
Treatment after failure	A patient who has previously been treated for TB and whose treatment failed at the end of their most recent course of treatment
Treatment after loss to follow-up	A patient who has previously been treated for TB and were declared lost to follow-up at end of their most recent course of treatment
Other preciously treated	A patient who has previously been treated for TB but whose outcome after their most recent course of treatment is unknown or undocumented.
Patient with unknown previous TB treatment history	A patient who has unknown previous TB treatment history

Classification and definition of the treatment results (applicable to Item (27) of the Page 1 of the form)

Classification	Susceptible TB	Resistant TB<br>(Multidrug resistant TB, extensively resistant TB and rifampin resistant TB)
Cured	A pulmonary TB patient with bacteriologically confirmed TB at the beginning of treatment who was culture-negative after the end of treatmet (or last month) and on at least one previous occasion	A patient who has completed a course of treatment without any sign of failure according to Korean guidelines and who is found to be negative in 3 or more culture examinations at intervals of at least 30 days after the intensive TB treatment phase
Completed	A TB patient who completed treatment without evidence of failure but with no record to show that sputum culture result after the end of treatment(or last month) on at least one previous occasion were negative, either the because tests were mot done or because result are unavailable.	A person who has completed the treatment according to the Korean quidelines but whose culture examination result does not meet the requirement of 'cured'
Failed	A TB patient whose sputum smear or culture if positive at month 5 or later during treatment.	A person who has finished the treatment due to one of the followings or needs the permanent change in prescription of at least 2 anti-TB drugs<br>- Failure in negative conversion after the intensive TB treatment phase<br>- Bacterial positive conversion during the maintenance phase<br>- Resistance to oral or parenteral quinolone is acquired additionally<br>- Side effects of TB drugs
lost to follow-up	The case in which the treastment is stopped for 2 months or longer in a row	
Died	The case in which a patient has expired before or during the treatment regardless of the cause of death	
Transfer-out	The case in which a patient is transferred out to other medical institutions without any sign of cured / completed / failed/defaulted	
Change in diagnosis	The case in which the person is diagnosed to have the other disease instead of TB	

The information on this form is used as critical data for the national TB monitoring system and the personal information is strictly protected. Thank you for cooperation.

How to notify and report the TB patients and how to enter the data in electronic data control system

[Personal profile of patient or dead person]

*(1) Name: Write down the name in Korean without any special sign or space. (In case of foreigner, write down the name described in the foreigner's ID card in English(including the space). If the foreigner's ID card doesn't exist, write down the name written on the passport in English(including the space).)

(2) Resident registration no.: Write down 13 numbers.
- ○ If the resident registration no. is entered, the sex and the age are automatically generated.
- ○ In case of foreigner, enter the foreigner's registration no. If the foreigner's registration no. doesn't exist, enter the date of birth and passport number written on the passport.
- ○ If unknown, enter it with the information as much as you know and then fill the unknown things with *.

*(3) Age: When the resident registration no. is entered, the age is automatically generated using the first 6 digits of the resident registration no. You can amend the automatically generated values. The age shall be filled in as it is the mandatory information.)

*(4) Sex: When the resident registration no. is entered, the sex is automatically generated using the first 6 digits of the resident registration no. (The information shall be filled in as it is the mandatory information.)

(5) Nationality: in case of foreigner, mark the check on the foreigner. Mark the nationality using the standard search tap of countries.

(6) Date of latest arrival: In case of foreigner, write down the date of latest arrival at Korea.

(9) Address: Enter the address written in the resident registration card of the patient (If unknown, write down the current residential loation.)

(10) Job: Mark the check mark on the appropriate one. If the patient has two or more job, mark all appropriate jobs with the mark.

(11)·(12) Fill it (11) name of facilities, (12) address of facilities such as company or school, and (10) job. If the patient have two or more jobs, write down all information for each job.

[Initial TB examination]

(13) Type of initial examination: Enter the information on the type of examination for diagnosis of the TB patient.
- ○ If the same examinations have been conducted two or more times (for example, TB cultures have been performed both in liquid and solid media), click "Add" to enter the information in the electronic data control system.

(14) Status and results of examination: Enter the state and results of the examination.
- ○ In case of chest X-ray test: mark the "TB suspected" on the "positive" blank and "Normal" in the "negative" blank.
- ○ In case of nontuberculous mycobacteria (NTM) on the examination of culture, mark it on the negative blank.

(16) Type of specimen: Classify it into the sputum and others.

[Medicine for diganosis and initial treatment]

(17) Code of disease: Fill it in using the TB code specified in Page 4 of the form. Fill it in down to the second decimal place(at least down to the first decimal place) for the classification of respiratory TB/ other TB and positive smear examination.

(18) Types of tuberculosis: Select the appropriate location of lesions from the electronic data control system using the search tab and enter it.

(19) Classification of patients: Fill it in using the classification and definition of TB patients (Page 2).
If the patient is transferred out to other medical institution, specify it on (30) special things.

(21) Medication: Write the generic names of the drugs initially prescribed by the medical institution after diagnosing the patient with TB.
- ○ Types and abbreviations of anti-TB durgs: isoniazid(H), rifampicin(R), ethambutol(E), pyrazinamide(Z), rifabutin(Rfb), kanamycin(Km), amikacin(Amk), capreomycin(Cm), streptomycin(S), levofloxacin(Lfx), moxifloxacin(Mfx), ofloxacin(Ofx), prothionamide(Pto), cycloserine(Cs), p-aminosalicylic acid(PAS), linezolid(Lzd), clarithromycin(Clr)

[Examination of resistance of TB drug]

Notify the required information (*) and the examination of the resistance to anti-TB drugs whenever the examination on the resistance to anti-TB drugs is performed. (In this case, click "ADD" on the electronic data control system before entering)

(25) Code of drug resistance TB: This is automatically selected when (24) the TB resistant drug is selected.
- ○ R = Rifampin resistant TB
- ○ H & R = U88.0(Multidrug-resistant TB)
- ○ H & R & (Lfx or Mfx or Ofx) & (Km or Amk or Cm) = U88.1(Extensively drug-resistant TB)

[Classification of the treatment results]

(27) Refer to the definition of the treatment results (specified in Page 2 of the form) and fill in the form.
In case of death, classify the cause of death into one of the two (TB related death or death due to others except for TB)

[Special things]

(30) Special to note: Fill in the actual residing address of patient, medicine used in the past, corrections and supplements and special notes which are critical other than the information for notification and reporting statement

The information on this form is used as critical data for the national TB monitoring system and the personal information is strictly protected. Thank you for cooperation.

Tuberculosis Code

Large classification	Middle classification	Narrow classification	description
A15			Respiratory tuberculosis, bacteriologically and histologically confirmed
	A15.0		Tuberculosis confirmed by sputum microscopy with or without culture
		A15.00	Tuberculosis of lung with cavitation, confirmed by sputum microscopy with or without culture
		A15.01	Tuberculosis of lung without cavitation or unspecified, confirmed by sputum microscopy with or without culture
	A15.1		Tuberculosis of lung, confirmed by culture only
	A15.2		Tuberculosis confirmed histologically
		A15.20	Tuberculosis of lung with cavitation, confirmed histologically
		A15.21	Tuberculosis of lung without cavitation or unspecified, confirmed histologically
	A15.3		Tuberculosis confirmed by unspecified means
		A15.30	Tuberculosis of lung with cavitation, confirmed by unspecified means
		A15.31	Tuberculosis of lung without cavitation or unspecified, confirmed by unspecified means
	A15.4		Tuberculosis of intrathoracic lymph nodes, confirmed bacteriologically and histologically
	A15.5		Tuberculosis of larynx, trachea and bronchus, confirmed bacteriologically and histologically
	A15.6		Tuberculous pleurisy, confirmed bacteriologically and histologically
	A15.7		Primary respiratory tuberculosis, confirmed bacteriologically and histologically
	A15.8		Other respiratory tuberculosis, confirmed bacteriologically and histologically
	A15.9		Respiratory tuberculosis, confirmed bacteriologically and histologically
		A15.90	Respiratory tuberculosis unspecified, with cavitation, confirmed bacteriologically and histologically
		A15.91	Respiratory tuberculosis unspecified, without cavitation or unspecified, confirmed bacteriologically and histologically
A16			Respiratory tuberculosis, not confirmed bacteriologically or histologically
	A16.0		Tuberculosis of lung, bacteriologically and histologically negative
	A16.1		Tuberculosis of lung, bacteriological and histological examination not done
		A16.10	Tuberculous of lung with cavitation, bacteriological and histological examination not done
		A16.11	Tuberculous of lung without cavitation or unspecified, bacteriological and histological examination not done
	A16.2		Tuberculosis of lung, without mention of bacteriological or histological confirmation
		A16.20	Tuberculosis of lung with cavitation, without mention of bacteriological or histological confirmation
		A16.21	Tuberculosis of lung without cavitation or unspecified, without mention of bacteriological or histological confirmation
	A16.3		Tuberculosis of intrathoracic lymph nodes,without mention of bacteriological or histological confirmation
	A16.4		Tuberculosis of larynx,trachea and bronchus, without mention of bacteriological or histological confirmation
	A16.5		Tuberculous pleurisy, without mention of bacteriological or histological confirmation
	A16.7		Primary respiratory tuberculosis without mention of bacteriological or histological confirmation
	A16.8		Other respiratory tuberculosis, without mention of bacteriological or histological confirmation
	A16.9		Respiratory tuberculosis unspecified, without mention of bacteriological or histological confirmation
		A16.90	Respiratory tuberculosis unspecified with cavitation, without mention of bacteriological or histological confirmation
		A16.91	Respiratory tuberculosis unspecified without cavitation or unspecified, without mention of bacteriological or histological confirmation
A17			Tuberculosis of nervous system
	A17.0		Tuberculosis meningitis
	A17.1		Meningeal tuberculoma
	A17.8		Other tuberculosis of nervous system
		A17.80	Tuberculoma of brain and spinal cord (Tuberculoma of brain and spinal cord)
		A17.81	Tuberculous meningoencephalitis
		A17.82	Tuberculous neuritis
		A17.88	Other tuberculosis of nervous system
	A17.9		Tuberculosis of nervous system, unspecified
A18			Tuberculosis of other organs
	A18.0		Tuberculosis of bones and joints
		A18.00	Tuberculosis of spine
		A18.01	Tuberculous arthritis of other joints
		A18.02	Tuberculosis of other bones
		A18.08	Tuberculosis of musculuarskeletal bones and joints, tuberculous synovitis, tuberculous tenosynovitis
	A18.1		Tuberculosis of genitourinary system
		A18.10	Tuberculosis of kidney and ureter
		A18.11	Tuberculosis of bladder
		A18.12	Tuberculosis of other urinary organs
		A18.13	Tuberculosis of prostate
		A18.14	Tuberculosis of other male genital organs
		A18.15	Tuberculosis of cervix
		A18.16	Tuberculous female pelvic inflammatory, tuberculous endometritis, tubrculous oophoritis and salpingitis
		A18.17	Tuberculosis of other female genital organs
		A18.19	Tuberculosis of genitourinary system, unspecified
	A18.2		Tuberculous peripheral lymphadenopathy
	A18.3		Tuberculosis of intestine, peritoneum, mesenteric lymph nodes
		A18.30	Tuberculous peritonitis
		A18.31	Tuberculous enteritis
		A18.32	Retroperitoneal tuberculosis
	A18.4		Tuberculosis of skin and subcutaneous tissue ·Erythema induratum, tuberculous
	A18.5		uberculosis of eye
	A18.6		Tuberculosis of ear ·Tuberculous otitis media
	A18.7		Tuberculosis of adrenal glands ·Addison's disease, tubeculous
	A18.8		Tuberculosis of other specified organs
		A18.80	Tuberculosis of thyroid gland
		A18.81	Tuberculosis of other endocrine glands
		A18.82	Tuberculosis of digestive tract organs, not elsewhere classified
		A18.83	Tuberculosis of heart, tuberculosis of myocardium, endocardium, pericardium
		A18.84	Tuberculosis of spleen
		A18.88	Tuberculosis of other sites
A19			Miliary tuberculosis
	A19.0		Acute miliary tuberculosis of a single specified site
	A19.1		Acute miliary tuberculosis of multiple sites
	A19.2		Acute miliary tuberculosis, unspecified
	A19.8		Other miliary tuberculosis
	A19.9		Miliary tuberculosis, unspecified
U88	U88.0		Multi-Drug resistant tuberculosis
	U88.1		Extensively drug-resistant Tuberculosis : XDR TB

Public Health Weekly Report, KCDC www.cdc.go.kr/phwr

ISSN : 2092-5964

Centers for Disease Control & Prevention

(28159) Osong Health Technology Administration Complex, 187, Osongsaengmyeong 2-ro, Osong-eup, Heungdeok-gu, Cheongju-si, Chungcheongbuk-do, Korea Center for Disease Control and prevention

TEL : 043-719-7341 FAX : 043-719-7339 http://www.cdc.go.kr
